# Molecular Markers of Diabetic Retinopathy: Potential Screening Tool of the Future?

**DOI:** 10.3389/fphys.2016.00200

**Published:** 2016-06-01

**Authors:** Priyia Pusparajah, Learn-Han Lee, Khalid Abdul Kadir

**Affiliations:** ^1^Jeffrey Cheah School of Medicine and Health Sciences, Monash University MalaysiaBandar Sunway, Malaysia; ^2^School of Pharmacy, Monash University MalaysiaBandar Sunway, Malaysia; ^3^Center of Health Outcomes Research and Therapeutic Safety (Cohorts), School of Pharmaceutical Sciences, University of PhayaoPhayao, Thailand

**Keywords:** diabetic retinopathy, biomarkers, screening, early stage retinopathy, personalized medicine

## Abstract

Diabetic retinopathy (DR) is among the leading causes of new onset blindness in adults. Effective treatment may delay the onset and progression of this disease provided it is diagnosed early. At present retinopathy can only be diagnosed via formal examination of the eye by a trained specialist, which limits the population that can be effectively screened. An easily accessible, reliable screening biomarker of diabetic retinopathy would be of tremendous benefit in detecting the population in need of further assessment and treatment. This review highlights specific biomarkers that show promise as screening markers to detect early diabetic retinopathy or even to detect patients at increased risk of DR at the time of diagnosis of diabetes. The pathobiology of DR is complex and multifactorial giving rise to a wide array of potential biomarkers. This review provides an overview of these pathways and looks at older markers such as advanced glycation end products (AGEs), inflammatory markers, vascular endothelial growth factor (VEGF) as well as other newer proteins with a role in the pathogenesis of DR including neuroprotective factors such as brain derived neurotrophic factor (BDNF) and Pigment Epithelium Derived Factor (PEDF); SA100A12, pentraxin 3, brain natriuretic peptide, apelin 3, and chemerin as well as various metabolites such as lipoprotein A, folate, and homocysteine. We also consider the possible role of proteins identified through proteomics work whose levels are altered in the sera of patients with DR as screening markers though their role in pathophysiology remains to be characterized. The role of microRNA as a promising new screening marker is also discussed.

## Introduction

Diabetes mellitus (DM) is associated with a wide range of microvascular complications including diabetic retinopathy (DR). One of the main risk factors associated with development of DR is poorly controlled blood sugar as assessed by glycated hemoglobin levels (HbA1c)—the higher the HbA1c, the greater the risk of developing retinopathy (Hiller et al., 1988). Legal blindness due to DR is estimated to be 25 times more common among the diabetic population than in those without diabetes (Aiello et al., 2001).

The range of treatments currently available for DM has dramatically increased the lifespan of diabetic patients, allowing time for clinically significant microvascular complications to develop. DR is currently estimated to be the leading cause of new onset blindness in working-aged adults in developed countries (Moss et al., 1998; Williams et al., 2004).

Based on clinical observations, it was initially assumed that microvascular complications only began to develop several years into the natural history of DM. However, in both the UKPDS and the Hoorn Study (Spijkerman et al., 2003), about 20% of the patients had microvascular diabetic complications including retinopathy, neuropathy, and proteinuria at the time of diagnosis. These findings raised the question of whether diabetes had been diagnosed late in this patient cohort or whether microvascular pathology actually develops during the early stages of DM.

There is now a large body of work showing that the pathological changes that eventually result in microvascular complications begin within days to weeks of onset of diabetes. Clinically, there is evidence of microvascular disease existing even in the pre-diabetic stage, albeit at a much lower prevalence than in those who are frankly diabetic (Ford et al., 2010). The Gutenberg study (Lamparter et al., 2014) revealed a prevalence of 8.2% for DR among the pre-diabetic population in Mid-Western Germany (7.2% had mild non-proliferative diabetic retinopathy (NPDR), 0.4% moderate NPDR and 0.2% had severe NPDR). This figure is in agreement with those from Shanghai where the prevalence of DR was 8% among pre-diabetics (Zhang et al., 2009) and also from the Diabetes Prevention Program in the USA where DR was detected in 7.9% of pre-diabetics (Nathan et al., 2007). The fact that DR can develop in the pre-diabetic stage suggests that even the lower levels of glycemia seen in pre-diabetes are detrimental to the retina.

Intensive treatment of diabetes may reduce the incidence and severity of DR as shown by the Diabetes Control and Complications Trial (DCCT) and UK Prospective Diabetes Study (UKPDS) studies (Diabetes Control Complications Trial (DCCT) Research Group, 1993; UK Prospective Diabetes Study Group, 1998). Recently in the FIELD study, it was suggested treatment of hyperlipidemia may reduce the progression of diabetic retinopathy and the need for laser treatment (Keech et al., 2007). However, in practical terms, it is difficult to provide intensive monitoring to all diabetics given the high prevalence of DM and the limited resources of any given healthcare system.

At present, effective treatments for DR exist and can prevent progression to blindness provided the disease is diagnosed early (Aiello et al., 2001). This highlights the need to develop effective screening methods for DR as identifying those at risk or detecting disease in its early stages is the key to preventing DR associated visual impairment.

Currently, formal diagnosis of retinopathy requires visualization of the retina which in turn requires specialized resources and specially trained staff. Given limited resources, there is a need for clinicians to identify the cohort at highest risk of developing DR and to prioritize screening of this category of patients. However, at present, there is a lack of accuracy and specificity in our ability to identify this particular cohort. There have been several studies analyzing clinical and demographic characteristics of diabetic patients to identify risk factors for DR—a systematic review identified duration of diabetes, hyperglycemia (as measured by HbA1c), hypertension, hyperlipidemia, pregnancy, and nephropathy/renal disease as consistent risk factors for diabetic retinopathy (Mohamed et al., 2007). Less consistent risk factors identified in this review were obesity, smoking, moderate alcohol consumption, and physical inactivity. While this information is of great clinical relevance, these factors will encompass a high percentage of patients and are not specific enough to truly narrow down the group most likely to have DR; particularly early stage DR.

At present DR is diagnosed by retinal examination. Performing retinal examinations on every diabetic patient represents an enormous logistical challenge, which is further compounded by the fact that the retina is a notoriously difficult area to assess accurately in a clinical setting. UK based studies show sensitivity levels of the detection of sight-threatening diabetic retinopathy of 41–67% for general practitioners, 48–82% for optometrists, 65% for ophthalmologists, and 27–67% for diabetologists and hospital physicians using direct ophthalmoscopy (Torok et al., 2013). Photographic methods currently use digital images with subsequent grading by trained individuals and have a sensitivity of 87–100% for the detection of sight-threatening retinopathy by trained personnel reading mydriatic 45° retinal photographs, with specificities of 83–96% (Torok et al., 2013). Utilizing photographic methods significantly increases the ability to correctly identify diabetic eye disease, however these cameras are costly and gaining access to centers with this equipment and trained personnel may be a challenge for the average patient population, especially in developing countries.

The subjectivity of direct eye assessment—even when performed by trained personnel—coupled with the limited access to fundal cameras, highlights the potential clinical benefit in identifying a biological marker that can accurately diagnose a patient with DR—particularly early DR—as this could not only be potentially more accurate but would also facilitate screening of a much wider segment of the population.

Advances in molecular medicine allowing for rapid identification of specific biomarkers may be the key to identifying those at risk of DR and facilitating detection of DR in its early stages thus allowing for timely intervention. This article aims to review the various molecular markers associated with the development of diabetic retinopathy. In particular we aim to focus on the molecules which show promise as screening tools, ergo have the capability to detect the disease in its early stages or, ideally, even before the actual onset of DR.

## Stages of diabetic retinopathy

DR can be classified into two stages: the non-proliferative phase (NPDR) and the more advanced proliferative phase (PDR). NPDR is characterized by changes in the retinal vessels i.e., microaneurysms, intraretinal hemorrhage, venous beading, and intraretinal microvascular abnormalities. NPDR is usually asymptomatic, however, left untreated it tends to progress to PDR which is often accompanied by deterioration in visual acuity. PDR is characterized by the proliferation of new vessels (neovascularization) believed to be triggered by retinal ischemia which then induces the release of growth factors including vascular endothelial growth factor (VEGF) (Giet et al., 2015). Bleeding and leakage from the unstable new vessels result in tissue alterations causing fibrovasular epiretinal membranes, vitreous hemorrhage, and tractional retinal detachment.

The other important component of DR is macular edema which is the most frequent complication of DR and the most common cause of vision loss due to diabetes (Cunha-Vaz et al., 2014). Macular edema is classified as mild, moderate, or severe based on the distance of the exudates and thickening from the center of the fovea (Wu et al., 2013).

## Anatomy of the retina and pathophysiology of diabetic retinopathy

### Normal anatomy of the retina

The retina is the neural layer of the eye, and is essentially an evagination of the brain consisting of layers of neurons and glial cells supplied by a rich vascular network from its dual blood supply from the choroid and the retinal vessels. The retina is among the most metabolically active tissues in the body making it highly susceptible to ischemic insults.

The retina contains photoreceptors—specially modified neurons—which receive light signals from the environment and convert to them to neural signals which are transmitted to the visual cortex of the brain via the optic nerve. One of the unique features of the retina is that it is the only neural tissue with direct exposure to light, making it susceptible to damage by photo-oxidized lipids which are highly toxic to the retinal cells (Simo and Hernandez, 2014).

The photoreceptors of the retina consist of two main types—the rods and the cones, with the cones being responsible for color vision. The macula has a very high density of cones and represents the portion of the retina which is most critical for fine vision and color vision; and as a result even minor damage to the macula—e.g., mild macular degeneration—can have significant impact on visual acuity. The photoreceptors and the supporting cells known as the glia represent the neural component of the retina, while the blood vessels that supply the retina represent the vascular component.

The blood vessels in the retina have unique features necessary to facilitate the normal functioning of the retina. The most apparent is the blood retinal barrier (BRB), a particularly tight and restrictive physiologic barrier that regulates ion, protein, and water flux into and out of the retina. The BRB consists of inner and outer components, the inner BRB being formed of tight junctions between retinal capillary endothelial cells and the outer BRB of tight junctions between retinal pigment epithelial cells. The BRB is essential to maintaining the eye as a privileged site and is essential for normal visual function (Cunha-Vaz et al., 2011). An additional unique feature of the retinal blood vessels is the high density of pericytes, cells which are critical for providing vascular stability, and control endothelial proliferation (Hammes et al., 2002).

### Overall pathophysiology of DR

**Figure 1:**
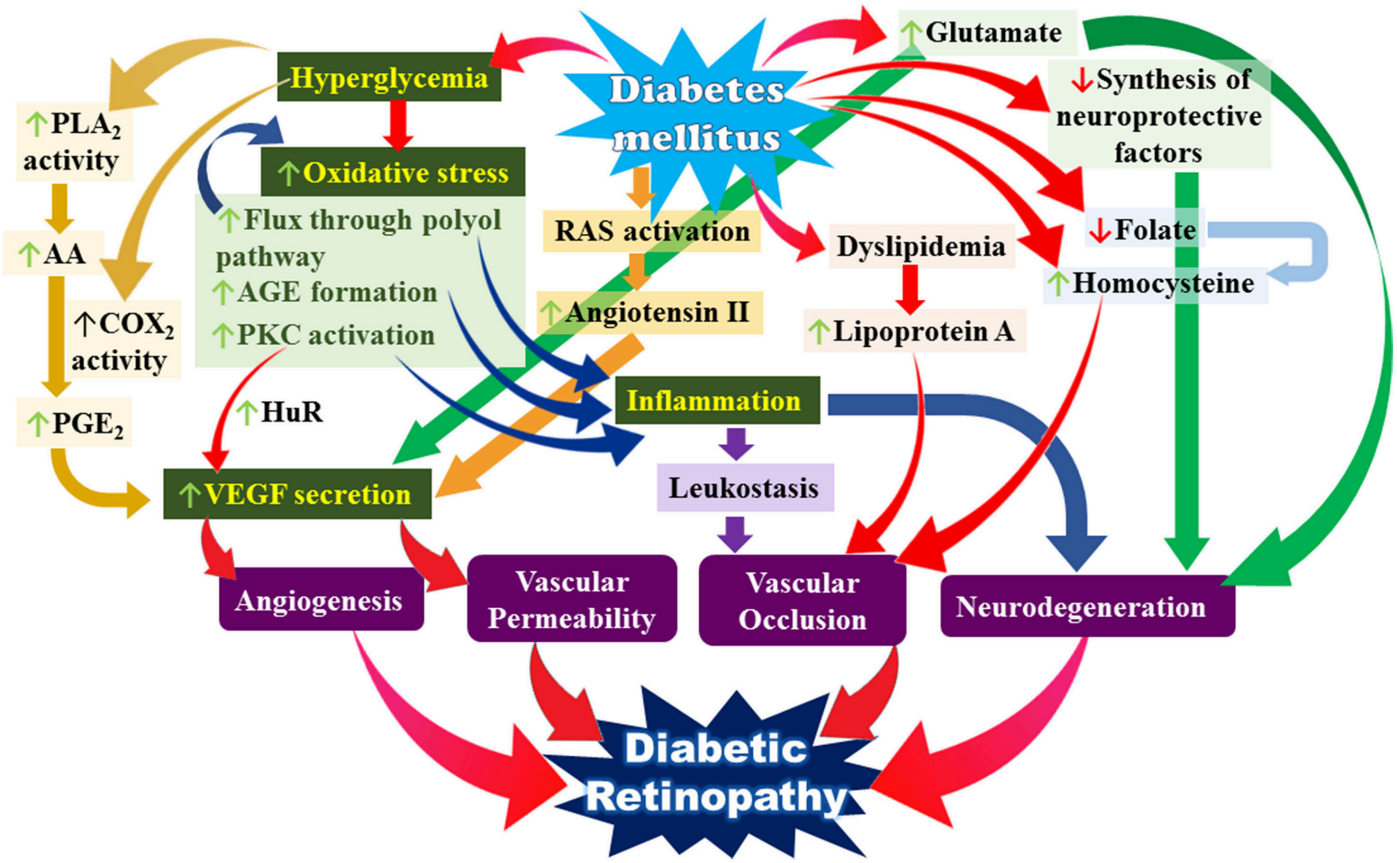
**Simplified overview of the multiple interacting pathways leading to the pathogenesis of diabetic retinopathy**. Key: PLA2, phospholipase A2; AA, arachidonic acid; COX2, cyclooxygenase 2; PGE2, prostaglandin E2; AGE, advanced glycation end-products; PKC, protein kinase C; VEGF, vascular endothelial growth factor; RAS, renin angiotensin system.

Our current understanding of the pathophysiology of DR suggests it is highly complex and multifactorial, involving the activation of several interrelated pathways which all tie in to several key mechanisms namely increased oxidative stress, increased pro-inflammatory mediators and increased VEGF secretion all occurring against a background of the various metabolic derangements that are inherent to DM (see Figure 1). DM also results in derangements in neurotransmitters and neuroprotective factors in the retina. The end result of all these events is damage to the neural and vascular components of the retina which eventually give rise to the clinical picture of DR.

There is evidence to suggest that diabetes results in impaired normal regulatory mechanisms throughout the neurovascular complex of the retina very early in the course of diabetes; before any apparent changes of NPDR are seen—as shown by reduced vasoconstriction in response to breathing 100% oxygen and decreased vasodilation in response to flickering light stimulation (Stem and Gardner, 2013).

Given that the clinical evidence of DR is predominantly vascular, the initial focus of work on DR focused on the vascular component; however it has been clearly demonstrated that the early changes affect the entire neurovascular bundle in totality, with both components contributing to functional loss. At present, there is enough information available to suggest that neural apoptosis precedes overt vascular abnormalities (Holopigian et al., 1992; Villarroel et al., 2010; Adams and Bearse, 2012; Reis et al., 2014). There is in fact emerging evidence that neurodegeneration participates in early microvascular changes that occur in DR such as breakdown of the BRB (via glutamate mediated excitotoxicity stimulating VEGF release), vasoregression, and impairment of neurovascular coupling (Simo and Hernandez, 2014).

Damage to the BRB is a key event in the early pathology of DR. The central mechanism of altered BRB function is a change in the permeability characteristics of retinal endothelial cells caused by elevated levels of growth factors, cytokines, advanced glycation end products, inflammation, hyperglycemia, and loss of pericytes (Klaassen et al., 2013).

The effect of all the various interacting pathways culminates in the eventual end points resulting in DR namely neurodegeneration, increased vascular permeability, vascular occlusion, and dysregulated angiogenesis. The neurodegeneration ultimately results in visual loss. While the initial triggers are directly a result of the metabolic derangements inherent to DM, chiefly hyperglycemia, the subsequent progression of pathology is related to the neuronal exposure to toxins crossing the damaged BRB and also ischemic damage as a result of impaired blood supply due to the vascular occlusion. Ischemia directly results in neuronal damage and death and the tissue non-perfusion also triggers the release of a multitude of growth factors which promote angiogenesis—the defining characteristic of PDR. The degree of cross talk between the interconnected elements giving rise to the overall picture of DR make it difficult to truly separate any one event from another.

Additionally, there appear to be additional factors affecting the pathogenesis of DR that remain to be defined as even diabetic patients with similar biochemical profiles may manifest a wide range of severity of microvasular complications. Cunha-Vaz et al. (2014) suggest that there are three different phenotypes of DR designated as A, B, and C where A is a slow progression type, whereas B and C are more aggressive with B being characterized by particularly high levels of leakage and C being characterized by signs of capillary closure. The mechanisms underlying the possible predominance of various pathways in different phenotypes remains to be defined.

Recently there has been great interest in exploring the role of microRNA (miRNA) in the etiology of DR. miRNAs are small, noncoding RNAs that represent a newly recognized, important level of gene-expression regulation that act post-transcriptionally to modulate expression of target genes via inhibition of protein expression by interfering with the translation and/or stability or mRNA (Cowan et al., 2014; Joglekar et al., 2016). To date, it is estimated that the entire human genome encodes for about 1100 miRNAs able to modulate the expression of about 60% of the protein-coding genes (Mastropasqua et al., 2014). miRNAs play important roles in diabetes and its complications as they regulate multiple biological pathways closely related to DR (Kovacs et al., 2011).

### Hyperglycemia and oxidative stress in DR

The key underlying element underpinning the core pathophysiology of DR appears to be hyperglycemia. The key role of hyperglycemia in triggering intracellular metabolic pathways that cause diabetic complications can also be inferred from the fact that diabetes selectively damages cells whose glucose transport rate does not decline rapidly as a result of hyperglycemia.

One of the key unifying mechanisms of hyperglycemia's effects is increased oxidative stress via increased superoxide production through the mitochondrial electron chain (Brownlee, 2005). This is believed to be the upstream event leading to increased flux through the polyol pathway, increased intracellular production of AGE precursors, increased PKC activation, and increased hexosamine pathway activity. Activation of these pathways then triggers increased production of pro-inflammatory substances through various other pathways. The effects of oxidative stress in diabetics are exacerbated by decreased levels of reducing substances—one of the mechanisms underlying this is increased flux through the polyol pathway which involves the NAPDH consuming generation of sorbitol from glucose via the action of aldose reductase, which in turn results in decreased levels of reduced glutathione rendering the cell more vulnerable to oxidative damage. Hyperglycemia mediated oxidative stress appears to be one of the linking factor between neurodegeneration and early microvascular abnormalities (Stem and Gardner, 2013).

Other pathways that have been implicated as potential links between hyperglycemia and DR include activation of growth factors such as vascular endothelial factor (VEGF) and insulin-like growth factor (IGF-1) and hemodynamic changes causing activation of the renin-aldosterone-angiotensin system (RAAS) (Tarr et al., 2013).

### Inflammation in DR

Current evidence suggests inflammation is one of the key players underpinning the pathological changes of DR. Chronic, low-grade subclinical inflammation is responsible for many of the signature vascular lesions of DR (Joussen et al., 2004). Clinical evidence proving this link was provided by the Hoorn Study, a population—based cohort study which recruited 625 patients and found that the prevalence of retinopathy was positively associated with tertiles of C reactive protein (CRP) and soluble intercellular cell adhesion molecule 1 (sICAM-1) (Van Hecke et al., 2005).

The characteristic low-grade chronic inflammation in diabetes is the result of the expression and production of numerous inflammatory markers including pro-inflammatory cytokines such as tumor necrosis factor alpha (TNFα), interleukin 1 (IL1), interleukin 6 (IL6), interleukin 8 (IL8), CRP, monocyte chemoattractant protein 1 (MCP1) as well as E-selectin and adhesion molecules such as intracellular adhesion molecule-1 (ICAM-1) and vascular cell adhesion molecule-1 (VCAM-1). Soluble forms of VCAM-1 and ICAM-1 designated sVCAM-1 and sICAM-1 are released from activated endothelial cells and control leukocyte activation and their migration to the site of inflammation. Elevated circulating and vitreous levels of sVCAM-1 have been demonstrated in patients with PDR (Kaul et al., 2010) and hyperglycemia has been shown to induce significant increase in expression of interleukin-1β (IL-1β), transmembrane receptors IL-1RI, and IL-RII as well as the natural antagonist receptor IL-1Ra (Scuderi et al., 2015). Reduced levels of lipoxin A4 (LXA4), a key mediator of the resolution of inflammation have been demonstrated in patients with DR (Kaviarasan et al., 2015). As illustrated in Figure 1, there are multiple pathways that lead to the increased production of these pro-inflammatory mediators.

One of the suggested mechanisms for the proinflammatory state in DM is the activation of toll like receptors (TLR) two and four which are activated by the hyperglycemia induced oxidative stress. It has been demonstrated that inflammation in hyperglycemic human retinal endothelial cells was attenuated by inhibition of TLR-4 and TLR-2 (Rajamani and Jialal, 2014).

A central and causal role of adherent leukocytes in vascular damage in DR—which is related to an increased expression of ICAM 1 and CD 18—results in increased numbers of leukocytes in retinal vasculature of diabetic humans and in animal work has been shown to begin as early as 1 week following experimental diabetes onset and results in injury to the endothelium via a Fas ligand (FasL)—mediated mechanism; a process which leads to breakdown of the BRB (Joussen et al., 2004). The presence of chronic inflammation also promotes increased production of VEGF which increases vascular permeability and later promotes angiogenesis.

Cyclooxygenase 2 (COX-2) is one of the key enzymes in the inflammatory cascade given its role in the production of prostaglandins (PGs) from arachidonic acid. COX-2 levels have been shown to be upregulated in diabetes via the PKC pathway following increased hyperglycemia-induced mitochondrial ROS production (Kiritoshi et al., 2003). An additional role for COX-2 as a modulator for angiogenesis via interaction with the VEGF pathway has been proposed with one possible route being an exciting new pathway, the ERK1-2/COX-2/PGE2 pathway which has been postulated as a signaling pathway mediating GPR-91 dependent VEGF release and thus contributing to the development of DR (Li et al., 2014).

Phopholipase A2 (PLA2), also a key enzyme in the inflammatory cascade, has been shown to be upregulated under conditions of hyperglycemia. Data indicates that high glucose directly damages pericytes through activation of PLA2/COX-2/VEGF-A pathway (Giurdanella et al., 2015). PLA2 releases arachidonic acid (AA) from phospholipids; with AA being the precursor of prostaglandins. Overall, data suggest that PLA2 are involved in BRB breakdown during the early stages of DR, by a mechanism involving the up-regulation of COXs, PG synthesis, VEGF, ICAM-1, and TNFα (Lupo et al., 2013).

Increased production of pro-inflammatory mediators is also triggered by interaction between Advanced Glycation End Products (AGE) and their receptors (R-AGE). The increased production of AGE in diabetics increases AGE-RAGE interaction which in turn activates the production of pro-inflammatory cytokines further contributing to the inflammatory state (Stem and Gardner, 2013).

### VEGF and its role in DR

VEGF is best known as a pro-angiogenic factor promoting the growth of new vessels in the proliferative phase of DR; and elevated VEGF levels in the vitreous are one of hallmark features of PDR. However, elevated levels of VEGF have been demonstrated in earlier stages of diabetic eye disease, suggesting that it may have other roles in the pathogenesis of DR. VEGF is currently thought to play a crucial role in pathogenesis of DR—causing breakdown of the blood-retinal barrier, stimulating endothelial cell growth and neovascularization, and increasing vascular permeability in the ischemic retina. The amount and duration of VEGF exposure required for BRB breakdown is less than that required for neovascularization (Jain et al., 2013). VEGF is also a proinflammatory molecule whose vitreal levels are highly correlated with retinal, neovascularization and edema (Tang and Kern, 2011).

There are many novel pathways leading to increased VEGF secretion which have been characterized providing new insight into the molecular mechanisms underlying the changes of DR which in turn provides exciting new possibilities for therapeutic targets as well as potentially pointing the way to new potential markers worth screening for to detect DR.

Amadio et al. (2010) described a PKCβ/HuR/VEGF pathway which is postulated to have a role in the pathophysiology of DR. PKC is a family of at least 10 serine-threonine kinases ubiquitously expressed and able to participate in multiple cellular functions. PKC β is the PKC isoenzyme preferentially activated in the eye. HuR is the ubiquitously expressed member of the ELAV (embryonic lethal abnormal vision) family, highly conserved mRNA binding proteins which act post-transcriptionally as positive regulators of gene expression which appears to protect VEGF mRNA from ribonucleases as well as enhancing its translation. This pathway was initially explored *in vitro* in retinal pericytes (Amadio et al., 2008) and subsequently in Sprague-Dawley rats (Amadio et al., 2010) and it was found that PKCβ/HuR activation was accompanied by enhanced VEGF protein expression that was blunted by a PKCβ inhibitor.

Genetic deletion of COX-2 diminished VEGF production in mouse retinal Muller cells (Yanni et al., 2010). One potential pathway by which this effect could be mediated is the recently described ERK1/2/COX-2/PGE2 pathway described earlier (Section Inflammation in DR).

An additional pathway increasing VEGF levels in DM could be the renin-angiotensin pathway which is upregulated in diabetic patients. Angiotensin II has been shown to have a positive effect on the secretion of VEGF (Wilkinson-Berka, 2006).

Ischemia is of course one of the strongest triggers for VEGF secretion and once vascular damage and occlusion lead to significant areas of tissue non-perfusion, this triggers increasingly elevated secretion of VEGF which then triggers angiogenesis.

### AGEs in pathology of DR

Advanced Glycation End Products (AGEs) are acknowledged to have a central role in the pathogenesis of the vascular complications of diabetes including DR (Kandarakis et al., 2014). AGEs are actually a complex group of compounds formed via a nonenzymatic reaction between reducing sugars and amine residues on proteins, lipids or nucleic acids. The role of AGEs in the pathobiology of DR has been demonstrated by vitreous work demonstrating increased levels in advanced disease (Nakamura et al., 2003; Pachydaki et al., 2006; Kakehashi et al., 2008). Glycation of proteins interferes with their normal functions by disrupting molecular conformation, altering enzymatic activity, and interfering with receptor functioning. AGEs form intra- and extra-cellular cross linking not only with proteins, but with some other endogenous key molecules including lipids and nucleic acids to contribute to the development of diabetic complications.

Hyperglycemia upregulates intracellular formation of AGEs, with the most abundant AGE present being carboxymethyllysine (CML) the levels of which have been suggested to be associated with incidence of diabetic complications (Li et al., 2012). Pentosidine is one of the other well-defined AGE products to date—it is synthesized through nonenzymatic reactions of pentose and its formation is closely related to oxidative processes. Increasing levels of plasma pentosidine have previously been linked to increased vascular rigidity of the retinal arteries in patients with type 2 diabetes with retinopathy (Sato et al., 2012).

In addition to the cross linking effect, AGEs exert their effects via binding to receptors for AGE (RAGE) which are expressed by several cells. AGE-RAGE interaction has clearly been demonstrated to be involved in the development of microvascular complications. However, in addition to cell bound RAGE, there are soluble forms in the plasma with soluble RAGE (sRAGE) representing a proteolytically cleaved form of RAGE, the role of which is much less well-defined. They may reflect the activity of the AGE-RAGE axis (Kerkeni et al., 2012) while other work suggests that they be inhibitors of AGE-RAGE mediated pathological effects (Grossin et al., 2008).

### Metabolic changes of diabetes and DR

Other links in the pathogenesis of DR related to metabolic derangements found in DM include elevated homocysteine (Malaguarnera et al., 2014) and lipoprotein a (Malaguarnera et al., 2013) levels. Reduced folate levels seen in diabetics have been found to be associated with an increased rate of DR (Malaguarnera et al., 2015). The pathways again are interlinked, however at present, reduced folate is believed to play a role via impaired nitric oxide production and impaired methylation ability resulting in DNA damage. The reduced folate levels also contribute to elevated homocysteine levels, where homocysteine is toxic to vascular endothelium and therefore induces thrombosis and contributes to ischemia.

DM is strongly associated with dyslipidemia and it has been demonstrated that atherogenic lipoproteins are associated with progression of retinopathy. Serum levels of lipoprotein (a) or Lp(a), an LDL like molecule, have been demonstrated to be elevated in patients with DR. One possible explanation for the connection between Lp(a) and DR is that Lp(a) induces a pro-thrombotic state by reducing fibrinolytic activity in the blood circulation (Malaguarnera et al., 2013).

### Altered levels of neurotransmitter and neuroprotective factors

The most important mechanisms in the neurodegenerative process are extracellular glutamate accumulation, oxidative stress (see Section Hyperglycemia and Oxidative Stress in DR), and reduction of neuroprotective factors synthesized by the retina.

Glutamate, the major excitatory neurotransmitter in the retina, has been found to be elevated in the extracellular space of the retina of experimental animals with diabetes. The glutamate excess results in excitotoxicity due overactivation of AMPA and NMDA receptors which then causes uncontrolled intracellular calcium response and ultimately cell death. Glutamate toxicity also contributes to glutathione depletion, contributing further to oxidative stress (Simo and Hernandez, 2014).

There are reports of reduced synthesis of several neuroprotective factors such as pigment epithelial-derived factor (PEDF), brain derived neurotrophic factor (BDNF), nerve growth factor (NGF), somatostatin (SST), and interstitial retinol-binding protein (IRBP) in the retina of diabetic patients compared with non-diabetic subjects (Stem and Gardner, 2013; Simo and Hernandez, 2014).

BDNF is a member of the neurotrophin family of growth factors and is important in the development, differentiation and maintenance of neurons. BDNF has been shown to inhibit apoptosis in rat retinal ganglion cells at early stages of DR and it has been shown that BDNF protects retinal neurons from hyperglycemia through the Tropomyosin-related kinase B (TrkB)/ERK/MAPK pathway (Liu et al., 2013).

Aside from its neuroprotective function, Pigment Epithelium-Derived Factor (PEDF) is also a potent anti-angiogenic factor (Dawson et al., 1999) with low PEDF levels predisposing to pathologic angiogenesis. Evidence comes from animal work in mice where treatment with PEDF inhibited retinal microvascular dysfunction (Longeras et al., 2012; Ibrahim et al., 2015) and enhanced survival of retinal ganglion cells (Unterlauft et al., 2014).

## Markers of diabetic retinopathy

There have been a large number of studies looking for a reliable biomarker of DR. Given that an ideal screening sample needs to be easily obtainable, blood markers (as opposed to vitreous) are the obvious choice as a blood sample is an easily obtained specimen with good acceptability rate among the typical patient population. Other samples that have studied with screening potential include skin biopsies and more recently tear fluid. This article does not aim to be an exhaustive review of all markers previously analyzed but focuses on those with greater potential as clinical markers of disease risk and progression. We therefore aimed to include work that looks at a spectrum of DR from mild to severe disease rather than those that only recruited patients with PDR. This was done as a useful screening marker would need to be a substance that shows elevated levels from early disease with a significant difference between the levels in diabetic patients without DR (NDR) and NPDR patients; an ideal marker should also show an increasing level with progressive disease severity as this would allow an estimation of disease severity and urgency of referral.

The studies selected for review have been divided into categories based on the substances analyzed namely AGEs, VEGF, inflammatory markers, and other proteins of various categories. The studies are summarized in Tables 1–**5**. Recent work looking for novel proteins via proteomics technology is also considered as well as miRNA which appears to have great promise as a biomarker. Although there is a fairly large body of work in the literature, it is largely limited by virtue of the fact that most are relatively small scale single center studies utilizing a cross sectional study design. Only two large scale prospective studies were found both of which were part of the work from the DCCT trial in the UK.

**Table 1:** **Summary of selected studies analyzing levels of AGEs in association with presence of varying stages of diabetic retinopathy**.

**Subtance(s) analyzed**	**Reference/study design**	**Patient population**	**Key findings**	**Method of analysis**
AGEs, sRAGE, and pentosidine (serum)	Kerkeni et al., 2012/cross sectional	*N* = 130 (40 NPDR, 60 PDR, 30 healthy controls)	Serum AGEs, sRAGE, and pentosidine levels significantly higher in patients with PDR vs. NPDR (*p* = 0.001, *p* = 0.01, and *p* = 0.005)	ELISA (Abo Switzerland Co. Ltd)
			no comparison against diabetics with no eye disease	
Furosine (glycated collaged) and CML (skin biopsy)	Genuth et al., 2005/prospective	*N* = 216 (65 NDR baseline, 57 with baseline mild to mod DR or microalbuminuria) + 40 age matched healthy controls	Furosine + CML predicted progression of retinopathy independent of HbA1c (χ^2^ = 59.4, *p* < 0.0001)	HPLC of prepared skin biopsy specimens
hydroimidazole (methylglyoxal derived AGE) (serum)	Fosmark et al., 2006/cross sectional	*N* = 227 (89 NPDR, 52 PDR, 86 no DR)	Serum levels of hydroimidizole higher in PDR vs. NDR (*p* = 0.002); significant increase in NPDR vs. NDR (*p* = 0.008)	Specific solid-phase, time-resolved competitive immunoassays (Delfia Wallac, Turku, Finland)
Nε-CML (serum)	Boehm et al., 2004/case control	*N* = 929 (81 NDR, 56 NPDR, 792 healthy controls)	Serum CML provided progressive risk marker for PDR (OR 24.5)independent of HbA1c; serum CML >1000 ng/ml strongly related to presence of clinically significant	Competition based ELISA (mouse monoclonal 4G9; Alteon, ramsey, NJ, USA)
			macular edema; serum CML levels elevated in DR vs. controls (*p* < 0.0001)	
N-epsilon-CML (Nε-CML) (serum)	Mishra et al., 2015/cross sectional	*N* = 80 (20 NDR, 20 NPDR, and 20 PDR, 20 healthy controls)	Mean levels of Nε-CML increased significantly with increasing severity of DR (*p* < 0.001 between controls, diabetics with no eye disease, NPDR, and PDR, respectively)	ELISA (Human Nε-CML ELISA kit from Uscn, Life Science Inc, USA)
N-epsilon-CML (Nε-CML) (serum)	Choudhuri et al., 2013/cross sectional	*N* = 379 (102 NDR, 77 NPDR, 105 PDR, 95 healthy controls)	Significant elevation of serum AGEs and Nε-CML in subjects with PDR (0 < 0.0001) and NPDR (*p* < 0.0001) compared to NDR	Nε-CML: ELISA (Cell Biolabs kit(catalog No STA 316)
AGEs (serum)				AGEs: ELISA(Cell Biolabs, SanDiego, CA) (kit no STA 317)
N-CML and pentosidine (serum)	Hirata and Kubo, 2004/cross sectional	*N* = 97 diabetic patients (42 NDR, 18 NPDR, 37 PDR)	Significantly higher blood levels of CML and pentosodine in PDR group compared to NDR (*p* < 0.01 and *p* < 0.05, respectively), no significant difference in CML or pentosidine levels between no DR and NPDR or between NPDR and PDR.	ELISA (assays prepared in own lab)
sRAGE and pentosidine (plasma)	Ng et al., 2013/cross sectional	*N* = 606 (171 NDR, 200 with DR (125 with NPDR; 75 with PDR), 235 healthy controls)	sRAGE/pentosidine ratio in DR patients was significantly lower than the ratio in diabetics without DR (*p* < 0.001); l higher levels of pentosidine, sRAGE, and sRAGE/pentosidine ratio in PDR compared to NPDR (*p* < 0.05, 0 < 0.01, and *P* < 0.01, respectively)	Standard ELISA sandwich kit: pentosidine (USCNK Life Scinece Inc, Wu Han, China); sRAGE (Biovendor Laboratorni Medicina akciova spolecnost, Brno, Reckovice, Czech Republic)

*CML, carboxymethyl-lysine; sRAGE, soluble receptors for advanced glycation end-products; NDR, diabetic with no retinopathy, NPDR, non-proliferative diabetic retinopathy; PDR, proliferative diabetic retinopathy*.

### Advanced glycation end-products (AGEs; Table 1)

As shown in Table 1, various AGEs have been analyzed as potential biomarkers of DR, with one of the key AGEs of interest being carboxymethyllysine (CML) which has been demonstrated to be elevated in the serum of patients with diabetic retinopathy (Boehm et al., 2004; Mishra et al., 2015). Significant differences were found in serum levels of N-epsilon carboxymethyl lysine between healthy controls and diabetics both with and without eye disease as well as increasing with progressive severity of DR.

Mishra et al. (2015) analyzed N-epsilon-CML (Nε- CML) in a total of 80 subjects—20 healthy controls and 60 diabetic patients (20 NDR, 20 with NPDR, 20 with PDR). Nε- CML levels increased with disease severity and were significantly different between all 4 groups (*p* < 0.001) and the levels also showed statistically significant correlation with the degree of disruption of the external limiting membrane (ELM) (*p* < 0.001). Univariate analysis with fasting blood glucose levels and HbA1c showed Nε- CML was an independent predictor of retinopathy. This seems to suggest it would be a good target for future work as it appears to show elevated levels from early disease compared to those without DR and increases with progressive disease severity allowing for estimation of urgency for treatment.

Mishra's findings were largely in agreement with earlier work which also found Nε- CML to be a promising marker of DR (Choudhuri et al., 2013). This study recruited a total of 379 subjects (95 healthy controls (HC), 102 NDR, 70 with NPDR, and 105 with PDR). They found that there was a statistically significant difference in serum Nε-CML levels between all four groups (HC vs. NDR *p* = 0.02; NDR vs. NPDR *p* < 0.001; NPDR vs. PDR *p* = 0.017). One intriguing finding here was that the level of Nε-CML was actually higher in NPDR compared to PDR and this was statistically significant. This was considered to be an indication that Nε-CML has pathogenic implications for retinal microvascular function in the earlier stages of DR. Choudhuri et al. (2013) also measured total serum AGE levels and though there was no significant difference between NPDR and PDR groups (*p* = −0.2643), there was a significant difference between the NDR and NPDR groups (*p* = 0.0297). This suggests that while serum AGE may not be a definitive marker of the severity of DR, it may be a promising marker of the presence of DR as an overall entity. In patients with NPDR and PDR there was a correlation between the serum levels of AGEs and Nε-CML, but this was not true in healthy controls and diabetics with no retinopathy.

Serum levels of Nε- CML in DR were also assessed in a case-control study which showed that high serum levels of CML were associated with proliferative retinopathy and clinically significant macular oedema (Boehm et al., 2004). This study had relatively few patients with eye disease but a very large cohort of controls whereby they recruited 56 patients with T2DM with DR, 81 diabetic patients with no DR, and 792 healthy controls. One limitation of this study from the perspective of potential screening markers is that even though the patients who were examined were classified according to ETDRS criteria into NPDR and PDR the results appear to have been analyzed collectively with the DR cohort consisting of both PDR and NPDR. The data from this trial showed that serum CML levels were significantly higher in DR group compared to controls (*p* < 0.0001) and that the levels were independent of HbA1c.

Prospective trials are always of particular interest and CML levels from skin biopsy were found to be a predictor of new occurence and progression of retinopathy in diabetic patients based on work by the DCCT team (Genuth et al., 2005). This trial was a prospective trial using patients enrolled in the DCCT study and showed that elevated levels of furosine (glycated collagen) and CML in skin and collagen were predictive of risk of future DR. This study looked at skin biopsies obtained from 211 patients in the DCCT trial ~1 year before the trial ended, and followed up this cohort for 10 years looking for development of or progression of existing retinopathy. In multivariate analyses, a combination of furosine and CML predicted the progression of retinopathy (χ^2^ = 59.4, *p* < 0.0001); even after adjustment for HbA1c (χ^2^ = 32.7, *p* < 0.0001). This trial also clearly demonstrated that the glycated proteins and AGE levels in skin was independent from HbA1c as a predictor of risk of progression of microvascular disease. The ability to predict those at high risk of disease even prior to disease onset would be make this an ideal marker to target those at increased need for intensive control and closer monitoring for complications.

Other studies assessing serum levels of methylglyoxal-derived hydroimidazole (an AGE) found the serum levels were raised in diabetic patients with retinopathy vs. those with no retinopathy (Fosmark et al., 2006). This cross sectional study recruited a reasonably large pool of patients—227 subjects with T2DM and various stages of DR (89 with NPDR, 62 with PDR and 86 NDR). The levels of serum hydroimidazole were on aggregate higher in the PDR compared to the NPDR group but the article did not attempt to analyze its ability to differentiate the different stages of DR. There was a statistically significant increase in hydroimidazole levels in DR compared to those with no DR (NPDR *p* = 0.008, PDR *p* = 0.002). While DR did correlate with HbA1c, the results showed that the serum levels of hydroimidazole were independent of the HbA1c. Multiple regression analysis showed a significant association between serum hydroimidazole levels and DR (OR = 1.45, *p* = 0.04). Given that there is a significant difference between controls and NPDR, this would also constitute a potential marker, however Nε-CML, having been found to be the prevalent AGE, appears to be a more promising target as a clinical marker.

Serum AGEs, sRAGE, and pentosidine levels in relation to severity of DR were analyzed in a study of 40 NPDR patients, 60 PDR patients, and 30 healthy controls (Kerkeni et al., 2012). However, this study did not include a group of diabetics with no eye disease making it difficult to comment on the ability of these substances to differentiate between a diabetic with and without eye disease. Overall the study found that all the substances analyzed showed significantly higher levels in PDR compared to NPDR, with AGE, sRAGE and pentosidine having significance levels of *p* = 0.001, *p* = 0.01, and *p* = 0.005, respectively.

Pentosidine has also been the focus of several studies, often in combination with CML. While it has been demonstrated to be increased in the serum of patients with DR, overall it appears less sensitive than CML in detection of DR (Hirata and Kubo, 2004; Kerkeni et al., 2012). Hirata and Kubo analyzed 97 diabetic patients—42 without eye disease (NDR), 18 with NPDR and 37 with PDR. CML and pentosidine levels were independent of HbA1c. There was no significant difference between either the CML or pentosidine levels between NDR and NPDR groups. The difference was only significant between NDR and PDR groups with *p* < 0.01 and *p* < 0.05 for CML and pentosidine, respectively. Again, this makes these markers less likely to detect early stage DR.

Recent work suggests that sRAGE/pentosidine ratio could be a risk factor determinant for DR (Ng et al., 2013). This study recruited 606 subjects (171 NDR, 125 with NPDR, 75 with PDR, and 235 healthy controls). The findings did not show a consistent elevation in either pentosidine in patients with DR versus diabetics without retinopathy. The levels of sRAGE were elevated in DR, and were statistically significant between combined NPDR and PDR against healthy diabetics without eye disease and PDR vs. NPDR but not for NPDR alone compared to NDR. When compared with diabetics without eye disease. sRAGE/pentosidine ratio was also analyzed and found to show a consistent upward trend with severity of DR, and a significant difference was found between all patients with DR even on multiple logistic regression (*p* = 0.048); however again there was no significant difference between NDR and NPDR groups.

Based on the work done so far, Nε-CML appears to be the AGE with the most potential as a screening marker for DR particularly with regard to being able to predict risk of developing DR in newly diagnosed diabetics.

### VEGF (Table 2)

VEGF is currently implicated as a mediator of NPDR and an initiator of PDR. VEGF has been widely studied and is postulated to have multiple roles in the pathogenesis of DR making it a logical target as a marker and the work done so far seems to support its potential in this light. A positive correlation between serum levels of VEGF and the incidence of DR (Ozturk et al., 2009) with the VEGF levels correlating with the stage of retinopathy (Cavusoglu et al., 2007) have been demonstrated.

**Table 2:** **Summary of selected studies analyzing levels of Vascular Endothelial Growth Factor (VEGF) in association with presence of varying stages of diabetic retinopathy**.

**Sample analyzed**	**Study design**	**Sample size**	**Key findings**	**Method of analysis**
VEGF (plasma)	Cavusoglu et al., 2007/Cross sectional	*N* = 83 (31 NPDR, 34 PDR,18 healthy controls)	VEGF levels increased significantly between NPDR and PDR (*P* = 0.016) and also between diabetics with NPDR vs. healthy controls (*p* < 0.000) as well as between PDR and healthy controls (*p* < 0.000)	ELISA (Biosource) sensitive to VEGF-165
VEGF (serum)	Du et al., 2014/Cross sectional (see also Table 4)	*N* = 69 diabetic patients (30 NDR, 23 NPDR, 16 PDR)	Increasing serum VEGF trend between no DR, NPDR and PDR groups; higher levels of VEGF in PDR vs. NPDR and NDR (*p* = 0.007 and *p* < 0.001, respectively); VEGF levels in NPDR vs. NDR also significantly higher (*p* = 0.007)	ELISA (human VEGF ELISA kit, Rapid Bio Lab, Calabasas, CA, USA)
VEGF (serum)	Jain et al., 2013/cross sectional	*N* = 77 (19 NDR, 19 NPDR, 20 PDR, 19 healthy controls)	VEGF levels were significantly different between the study groups (*p* < 0.001) by ANOVA	Human VEGF ELISA kit, Invitrogen
VEGF (serum)	Ozturk et al., 2009/cross sectional	*N* = 156 (31 NDR, 49 NPDR, 46 PDR, 28 healthy controls)	VEGF levels higher in those with NPDR compared to controls (*p* = 0.01), and in PDR compared to controls (*p* = 0.02). No significant difference between NPDR and PDR (*p* = 0.87)	Luminex multiplex bead immunoassay (Human Cytokine LINCOplex kit; LINCO Research, St Charles, MO)

*VEGF, Vascular Endothelial Growth Factor; NDR, diabetic with no retinopathy; NPDR, non-proliferative diabetic retinopathy; PDR, proliferative diabetic retinopathy*.

A cross sectional analysis of 69 diabetic patients (30 NDR, 23 NPDR, and 16 PDR), were able to show an increasing serum VEGF trend with increasing severity of DR, with a statistically significant difference between NDR vs. NPDR (*p* = 0.007) which would suggest it does have potential as a screening marker. (Du et al., 2014) However, these findings are not consistently replicated by the other studies in the literature.

Jain et al. (2013) analyzed serum VEGF levels in 19 patients with NPDR and 20 patients with PDR and compared them against 19 diabetics with no eye disease and 19 healthy controls. There was a significant elevation in VEGF with progressive severity of retinopathy (OR 3.98, CI 95%). ANOVA showed statistically significant differences between all groups (*p* < 0.001) however further analysis using Tukey's multiple comparisons showed significant differences only between control and NPDR, controls and PDR, and no DR and PDR. The lack of significance between no DR and NPDR groups seems to cast some doubt on its potential as a screening marker of early disease.

Ozturk et al. (2009) performed a cross sectional study with 49 patients with NPDR and 46 with PDR vs. 31 diabetics without DR and 28 normal controls and their findings were that the median serum VEGF level showed a statistically significant difference (*p* > 0.05) between all groups except between NPDR and PDR. The finding of significance between NDR and NPDR agree with Du et al. (2014) but contradict the findings of Jain et al. (2013), however the relatively small sample sizes as well as geographic variation in the patient population may account for this.

Cavusoglu's et al. (2007) findings were also from cross sectional data looking at 31 patients with NPDR, 34 patients with PDR and 18 healthy controls. VEGF levels increased significantly between those with NPDR and PDR (*p* < 0.016) however they did not include a group of diabetics without eye disease as comparison for the VEGF levels which limits the ability to comment on its ability as a screening marker in this particular study.

Although each individual study above had a relatively small number of patients with eye disease in each cohort the consistent finding of a statistically significantly increasing serum or plasma level of VEGF with increasing disease severity was notable; making it a potentially viable clinical marker for DR presence and severity.

### Inflammatory markers (Table 3)

While it clear that inflammation is key to the pathophysiology of DR, inflammation is also strongly associated with the other microvascular complications of DR, suggesting that use of markers of inflammation alone may not be sufficient as a unique marker of DR. Perhaps further work may elucidate particular isoforms unique to retinal pathology which may be more specific as screening markers.

**Table 3:** **Summary of selected studies analyzing levels of inflammatory markers in association with presence of varying stages of diabetic retinopathy**.

**Substance(s) analyzed**	**Reference/study design**	**Patient population**	**Key findings**	**Method of analysis**
α2- anti plasmin, fibrinogen, plasminogen, PAI-1 (plasma)	Polat et al., 2014/cross sectional	*N* = 52 (21 NDR, 18 NPDR, 13 PDR, 40 healthy controls)	Significantly elevated levels of α2-anti plasmin in diabetics, with significant elevation with increasing severity of eye disease: *p* < 0.005 for NPDR and *p* < 0.001 for PDR	α2-anti plasmin, plasminogen:ELISA (Cusabio Biotech Co Ltd)
			PAI-1 levels were higher in diabetics compared to non-diabetics but did not reach significance (*p* = 0.209)	PAI-1: ELISA (Border Med System, Vienna)
			Fibrinogen and plasminogen levels were similar between diabetics and controls.	Fibrinogen: ELISA (MTI Tokyo)
hs CRP (serum)	Sasongko et al., 2014/cross sectional	*N* = 224 (23 NDR, 144 with mild to moderate NPDR (non-vision threatening), 57 with severe NPDR or PDR (vision threatening)	Statistically significant increase in hsCRP level in vision threatening vs. non-vision threatening DR (OR 1.3 in multiple regression model, 95% CI 1.1-1.5)	hsCRP: Nephelometry (Nephelometer; Siemens Healthcare Diagnostics Inc, Newart, DE, USA)
sICAM-1, VCAM-1,E-selectin, endothelin -1, total nitrite			No correlation between serum markers of endothelial function and DR severity	All others: ELISA (R&D systems, Minneapolis, MN, USA)
ICAM-1 (intercellular adhesion molecule-1) (serum)	Jain et al., 2013/cross sectional	*N* = 77 (19 NDR, 19 NPDR, 20 PDR, 19 healthy controls)	ICAM-1 levels were significantly different between the study groups (*p* < 0.001)	Human sICAM-1 ELISA kit, Invitrogen
NO, sIL2R, IL 8 and TNF α (serum)	Doganay et al., 2002/cross sectional	*N* = 67 (15 NDR, 18 NPDR, 19 PDR, 15 healthy controls)	Statistically significant elevation of NO, sIL2R, IL 8, and TNF alpha between NPDR vs. no DR and controls (*p* < 0.01); and also between PDR vs. NPDR, no DR, and controls (*p* < 0.001). no statistically significant difference between controls and DM with no DR (*p* > 0.05)	Cytokines and chemokines: Chemiluminescent immunometric assay (Immulite, Diagnostic Products, Los Angeles); NO via spectrophotometric quantitation using Griess reagent
PTX3, hsCRP (plasma)	Yang et al., 2014/case-control	*N* = 163 (30 NDR, 28 mild NPDR, 21 moderate NPDR, 23 severe NPDR, 20 PDR, 41 healthy controls)	Proportion of higher-degree retinal complications increased in direct correlation with log PTX3 levels (*p* trend < 0.001) vs. log hs-CRP-values *P* trend < 0.006. ROC curves for PTX3 show diagnostic sensitivity for DR 53.3%, specifiticy 91.7 vs. 51.1% and 70.8% for hs-CRP)	PTX3: ELISA (R&D Systems IC, Minneapolis, MN, USA); hs CRP; ELISA (DakoCytomation, Copenhagen, Denmark; human hsCPR standards from Randox Laboratories, Count Antrim, UK)
RANTES, SDF-1α (serum)	Meleth et al., 2005/cross sectional	*N* = 93 (62 less severe DR, 31 severe NPDR or worse)	Significant elevation between at least severe NPDR vs. less severe DR: RANTES (*p* < 0.001) and SDF-1α (*p* < 0.007)	ELISA (R&D Systems Inc, Minneapolis, MN)
sE-selectin, PAI 1 (serum)	Rajab et al., 2015/prospective	*N* = 1391 diabetic patients with either mild or moderate retinopathy or no DR at baseline (260 progressed to severe NPDR and 831 progressed three steps in severity of DR)	High levels of sE selectin and PAI 1 at baseline are associated with development of retinopathy in patents who had no retinopathy at baseline. Increased levels of PAI 1 correlated with risk of progression to severe pre-proliferative or PDR	Signature Plus Protein Array imaging and Analysis System (Aushon BioSystems) uwing Array VisionTM software for data analysis

*NO, nitric oxide; sICAM-1, soluble intercell adhesion molecule-1; VCAM-1, vascular cell adhesion molecule-1; sIL2R, Soluble interleukin-2 receptor; IL-8, interleukin-8; tumor necrosis factor alpha (TNF α); RANTES, Regulated on Activation, Normal T-cell Expressed and Secreted; SDF-1α, stromal derived factor-1α; sE-selectin, soluble-E-selectin; PAI 1, plasminogen activator inhibitor 1; hs CRP, high sensitivity C-reactive protein; PTX 3, pentraxin 3; NDR, diabetic with no retinopathy; NPDR, non-proliferative diabetic retinopathy; PDR, proliferative diabetic retinopathy; ROC, receiver operating curve*.

Among all the studies reviewed, perhaps the most significant is the work done as a follow up to the DCCT trial which was a prospective trial with a large patient cohort. One of very few prospective studies found, this work highlighted the predictive role of markers of inflammation and endothelial dysfunction on the course of DR. A cohort of 1391 type 1 diabetics with either mild or moderate retinopathy or albuminuria or completely free of complications were followed up and carefully assessed over a period of 16–20 years for 3-step progression from baseline in retinopathy score; and the results show that high levels of soluble E-selectin (sE-selectin) and Plasminogen activator inhibitor 1 (PAI 1) at baseline are associated with development of retinopathy in patients who had no retinopathy at baseline. Increased levels of PAI 1 also correlated with risk of progression to severe pre-proliferative or proliferative DR (Rajab et al., 2015). This suggests that these markers of endothelial dysfunction and decreased fibrinolysis may be indicative of retinopathy development and progression. Another key finding in this study was that while conventional inflammation markers did show elevated levels in patients with retinopathy, they had no predictive value for the development/progression of retinopathy. This suggests that conventional markers of inflammation may enable the presence of existing retinopathy to be identified but cannot predict retinopathy progression or development.

Sasongko et al. (2014) analyzed serum hsCRP in 224 subjects—24 NDR, 144 with mild to moderate NPDR (defined as non-vision threatening) and 57 with severe NPDR or PDR (defined as vision-threatening). The study found that in vision threatening vs. non-vision threatening DR, there was a statistically significant increase in serum hsCRP level in a multiple regression model (OR 1.3; 95% CI 1.1–1.5). The association was more prominent in patients with a BMI ≥ 30 kg/m2 (OR 2.7 vs. OR = 1.7 for non-obese group at same hsCRP level). However, again, there was no significant difference between NDR and non-vision threatening DR. This study also measured the levels of sICAM-1, VCAM-1, E-selectin, endothelin-1, and total nitrite but none of these shown a correlation with DR.

It has recently also been found that plasma levels of pentraxin 3 (PTX3, an acute phase reactant which reflects impaired vascular endothelial function) are associated with the development and progression of DR in Korean patients with Type 2 diabetes mellitus (Yang et al., 2014). This was a case-control study which recruited 163 subjects—92 diabetic patients with DR, 30 diabetics with no DR and 41 healthy controls where plasma levels of PTX3 and hsCRP were measured and compared. Of the 92 patients with DR, 28 had mild NPDR, 21 had moderate NPDR, 23 had severe NPDR, and 20 had PDR. The proportion of higher-degree retinal complications increased in direct correlation with log PTX3 levels with a *P* trend < 0.001 whereas a similar analysis based on log hsCRP values had a *P* trend of 0.006. Based on PTX3 and hsCRP levels selected based on receiver operating curves, the diagnostic sensitivity of PTX3 for DR was 53.3% and sensitivity 91.7% while for hsCRP it was 51.1 and 70.8%, respectively. The authors therefore suggested that PTX3 may be a more accurate predictor of DR development than hsCRP. The presence of elevated PTX3 levels from early disease and its progressive elevation with increasing disease severity seem to suggest it has potential as a screening marker.

Serum α2 antiplasmin is an additional inflammatory marker which has been shown to increase with increasing disease severity. A recent cross sectional study showed a significant difference between serum α2 antiplasmin in NDR and DR patients and, of particular note, it showed significantly elevated levels even in early eye disease (*p* < 0.05 for NPDR and *p* < 0.001 for PDR in comparison to NDR; Polat et al., 2014). This study enrolled 52 diabetic patients (21 without DR, 18 with NPDR, and 13 with PDR) and 40 healthy controls. PAI-1 was found to be elevated in patients with DR but did not reach significance. The true importance of these markers may be their role in predicting progression or development of DR rather than detecting its presence as suggested by Rajab et al. (2015).

The mean serum nitric oxide (NO), soluble interleukin-2 receptor (sIL2R), interleukin- 8 (IL-8), and tumor-necrosis factor-alpha (TNF-alpha) levels have been shown to increase with the stage of DR with the highest levels being found in patients with PDR (Doganay et al., 2002). This was a cross sectional study which recruited 19 patients with PDR, 18 with NPDR, and 16 diabetics with no retinopathy as well as 15 healthy controls. The levels of NO, sIL2R, and TNF-alpha showed a statistically significant increase for PDR patients compared to that of NPDR, no DR and controls (*p* < 0.001) as well as for NPDR vs. no DR and controls (*p* < 0.01). There was no statistically significant difference in serum levels of these markers between diabetic patients without eye disease compared to controls (*p* > 0.05). This was one of very few studies that was able to demonstrate a significant rise in non-specific inflammatory markers with DR, and the strength of the findings is limited by the small sample size.

Regulated on Activation, Normal T-cell Expressed and Secreted (RANTES) and stromal derived factor—1α (SDF-1α) were shown to be significantly elevated (*p* < 0.001 for RANTES and *p* < 0.007 for SDF-1α) in patients with at least severe NPDR compared with those with less severe DR (Meleth et al., 2005). This cross sectional study analyzed serum from 93 patients—62 with less severe forms of DR and 31 patients with severe NPDR or worse. However, drawing more definite conclusions about their potential as screening tools is limited by the lack of healthy controls and diabetics with no eye disease in this study. Some of the studies reviewed also included various inflammatory markers in the panels of substances analyzed but found limited correlation with DR (Dong et al., 2015; Kaviarasan et al., 2015). These are included in the relevant tables.

Overall, most of the inflammatory markers reviewed lack the ability to consistently detect DR in its early stages—they may be of more use in aiding determination of disease severity rather than diagnosing new disease. However, Rajab's work suggests that certain markers may be of predictive value which would make them excellent screening targets particularly in newly diagnosed diabetics.

### Other markers (Table 4)

A wide variety of additional markers have been studied. However, most of these markers have only been the subject of one or at the most two relatively small studies, making it difficult to make any definitive conclusions about their true efficacy as screening markers.

**Table 4:** **Summary of selected studies analyzing levels of other markers in association with presence of varying stages of diabetic retinopathy**.

**Substance analyzed**	**Reference/Study method**	**Patient population**	**Key findings**	**Method of analysis**
Apelin 13 (serum)	Du et al., 2014 /cross sectional (see also Table 2)	*N* = 69 (30 NDR, 23 NPDR, 16 PDR)	Significant elevation in serum apelin between PDR and no DR (*p* = 0.041); no significant difference in levels between NPDR and no DR group	ELISA (human apelin-13 ELISA kit; Uscnlife Science and Technology Company, Missouri)
BDNF (serum)	Kaviarasan et al., 2015/cross sectional	*N* = 114 (27 NDR, 30 NPDR, 30 PDR and 27 healthy controls)	Significantly lower serum in both NPDR and PDR compared to healthy controls for BDNF (*p* = 0.0071, *p* = 0.0075)and LXA4 (*p* = 0.020, *p* = 0.008); BDNF and LXA4 levels shows progressive drop as seen in median across no DR, NPDR, and DR groups.	BDNF: ELISA (Chemikine)
LXA4 (plasma)			IL-6 significantly increased in NPDR and PDR compared to healthy controls. Other cytokines no significant increase.	LXA4: ELISA (Oxford)
IFN-γ, TNF-α, IL-10, IL-6.IL-4, IL-2				Cytokines: cytometric bead array system (BD Biosciences, Germany)
Chemerin (serum)	Du et al., 2016/cross sectional	*N* = 80 (25 NDR, 20 NPDR, 15 PDR, 20 healthy controls)	Serum chemerin levels showed statistically significant increase with increasingly severe eye disease; trend chi square for chemerin level vs. sensitivity of DR χ^2^ = 16.07, *p* < 0.001	ELISA (human Chemerin ELISA kit, Uscnlife Science and Technology Company, Missouri, TX)
Lipoprotein (a) [Lp(a)] (Serum)	Malaguarnera et al., 2013/cross sectional	*N* = 145 (78 NDR, 67 DR)	Elevated Lp(a) levels were found in 78.3% of patients with DR but only in 21.75% of diabetic patients with no DR	Immunonephelometry (Olympus AU640 Medican Watford, UK)
Folate (plasma, red cell folate)	Malaguarnera et al., 2015/cross sectional	*N* = 231 (96 NDR, 70 NPDR, 65 PDR)	Severity of DR associated with lower folic acid and red cell folate levels with a significant difference between PDR and NPDR (*p* < 0.05)	Folate measured with Quantaphase II folate radioassay kit (Bio-Rad Laboratories, Hercules, CA, USA)
Homocysteine (plasma)			Higher plasma levels of homocysteine in NPDR and PDR compared to NDR, respectively (*p* < 0.001 in both)	Homocysteine measured with immunoassay
Homocysteine (serum)	Malaguarnera et al., 2014/cross sectional	*N* = 330 (50 NDR, 63 NPDR, 62 PDR, 80 healthy controls, 75 randomly selected patients)	Homocysteine levels were significantly elevated between groups with progressive elevation of levels with worsening retinopathy; significant elevation between controls and NDR (*p* < 0.001), NDR vs. NPDR (*p* < 0.001) and NPDR vs. PDR (*p* < 0.001)	Measured using the method of Asaki and Sako
NT-proBNP (serum)	Hamano et al. (2014)/cross sectional	*N* = 277 (60 diabetics with no vascular complications and 217 with micro or macrovascular complications—out of these 217, 74 had DR, 83.7% with NPDR and 16.3% with PDR)	Odds ratio of having retinopathy was 13.78 in patients with NT-proBNP in the highest tertile independent of age, sex, duration or diabetes, HbA1c and BMI.	Two-site sandwich electrochemiluminescence immunoassay (ECLusys proBNP; Hoffman-La Roche Ltd, Basel, Switzerland)
PEDF (plasma)	Ogata et al., 2007/cross sectional	*N* = 145 (12 NDR, 16 NPDR, 39 severe NPDR, 45 PDR, 33 healthy controls)	Diabetic patients had significantly higher plasma PEDF vs. healthy controls (*p* = 0.03), statistically significant rise in plasma PEDF in PDR vs. healthy controls (*p* = 0.005); no significant difference between the other diabetic retinopathy groups.	ELISA Kit (Chemikine PEDF Sandwich ELISA Kit; Chemicon Internation, Temecula, CA)
BDNF	Liu et al., 2016/cross sectional	*N* = 344 (251 NDR, 46 non-vision threatening DR, 47 vision threatening DR)	Plasma BDNF was significantly lower in diabetics with DR compared to those without DR (*p* < 0.001) with adjusted OR 0.79 (95% CI, 0.73–0.85); also for diabetics with VTDR compared to all NDR and those with non-VTDR (*p* < 0.001) and adjusted OR 0.61 (95% CI, 0.57–0.70)	BDNF: sandwich ELISA kit (DuoSet ELISA Development, R&D Systems, Inc USA)
S100A12 @calgranulin C @ EN-RAGE (plasma)	Dong et al., 2015/cross sectional (with respect to eye markers; prospectively followed for occurrence of macrovascular events)	*N* = 372 (113 NDR, 42 mild NPDR, 35 moderate NPDR, 38 severe NPDR, 36 PDR, 108 healthy controls)	Plasma levels of SA10012 independently associated with presence of DR in pts with T2DM (odds ratio 1.421, confidence interval 1.036-2.531; AUC of ROC curves using log SA100A12 0.822 (*p* < 0.001)	S100A12 and pentosidine: ELISA (Cusabio, Wuhan, China)
hsCRP, sRAGE, pentosidine			No significant correlation with hsCRP, pentosidine or sRAGE levels	hsCRP: at Clincal Diagnostic Laboratory
				sRAGE: ELISA(Biovendor Laboratorni Medicina)

*PEDF, pigment epithelium derived factor; BDNF, brain derived neurotrophic factor; LXA4, Lipoxin A4; hsCRP, high sensitivity C reactive protein; IL-6, interleukin 6; IFNγ, Interferon Gamma; TNF- α, Tunour Necrosis Factor alpha; sRAGE, soluble receptor for advanced glycation end-products; NDR, diabetic with no retinopathy; NPDR, no proliferative diabetic retinopathy; PDR, proliferative diabetic retinopathy, VTDR, vision threatening diabetic retinopathy*.

Among the studies reviewed, one of the more promising markers of early DR appears to be S100A12, a calcium binding proinflammatory protein also known as calgranulin C or extracellular newly identified receptor for AGE binding protein (EN-RAGE) (Dong et al., 2015). This molecule (a calcium binding pro-inflammatory protein) appears to have promise as a marker of DR as it was shown to be independently associated with DR by Dong et al. (2015) who performed a cross sectional study of markers of DR with 372 subjects (113 NDR, 42 mild NPDR, 35 moderate NPDR, 38 severe NPDR, 36 PDR, and 108 healthy controls). They found that plasma S100A12 levels were independently associated with the presence of DR in patients with T2DM (odds ratio 1.421, 95% CI 1.036–2.531, *p* = 0.033). LogS100A12 values showed significant differences between groups with progressive severity of DR. Receiver operating curves (ROC) of log S100A12 concentration in the plasma were able to select a value of 90.16ng/mL to provide a diagnostic sensitivity and specificity for DR development of 78.1 and 77.0%, respectively; area under the curve was 0.822 (*p* < 0.001) suggesting that plasma levels of S100A12 might be a promising predictive biomarker of DR. On follow up of these patients, SA100A12 at baseline also appeared to correlate with occurrence of macrovascular events. The levels of hs-CRP, pentosidine, and sRAGE were also analyzed but did not show significant correlation with DR severity.

Serum levels of chemerin, a multifunctional peptide involved in lipid and glucose metabolism (Fatima et al., 2015) has also been found to be elevated in patients with NPDR and PDR (Du et al., 2016). This study analyzed 60 T2DM patients (15 with PDR, 20 with NPDR, and 25 with no DR) and 20 healthy controls. Among the diabetic patients the serum chemerin levels were demonstrated to increase with increasingly severe eye disease; the difference in chemerin was statistically significant (*p* < 0.05) between the three groups. A trend chi-square showed that the chemerin level was correlated with the severity of DR (χ^2^ = 16.07, *p* < 0.001). In addition, levels of chemerin were also found to correlate positively with levels of other markers associated with markers of obesity, inflammation, and neovascularization namely CRP and VEGF. While these findings are promising, the significance of this study is limited by the small patient numbers.

Apelin-13, a ligand of G-protein coupled receptor which has been shown to be involved in retinal angiogenesis was also targeted as a potential biomarker (Du et al., 2014). This study recruited 69 type 2 diabetics, 16 with PDR, 23 with NPDR, and 30 with NDR. Serum levels of apelin-13 were significantly elevated in the PDR group compared to the no DR group (*p* = 0.041). However, there was no significant difference in apelin-13 levels between the NPDR and NDR group limiting its promise as a screening marker.

Recent work in Japan also suggest that N-terminal fragment on probrain natriuretic peptide (NT-proBNP) is associated with comorbid diabetic microvascular complications with a strong correlation for DR (Hamano et al., 2014). This was a cross sectional study which recruited 277 subjects (60 diabetics with no vascular complications and 217 with micro or macrovascular complications—out of these 217, 74 had DR, 83.7% with NPDR, and 16.3% with PDR). The odds ratio for DR was found to be 13.78 (95% CI 3.34–70.75) in the highest tertile of NT-proBNP compared to the lowest tertile; and the elevation of this marker in DR was independent of disease duration in addition to kidney function or age. However, as the DR patients appear to have been analyzed as a single group rather than by severity of DR it is difficult to gauge whether NT-proBNP may be a potential marker for early retinopathy.

While PEDF levels have been found to be reduced in the vitreous in DR (Ogata et al., 2002; Boehm et al., 2003), serum levels have been demonstrated to be elevated in type 1 (Jenkins et al., 2007) and type 2 diabetes (Jenkins et al., 2008) with a correlation to microvascular complications in type 1 diabetes; but with no specific association with DR. Other work showed higher levels of plasma PEDF in patients with PDR Ogata et al., 2007). This study recruited 145 patients—112 with T2DM (no DR = 12, mild to moderate NPDR = 16, severe NPDR = 39, PDR = 45) and 33 healthy controls. Overall, patients with diabetes were found to have a higher plasma PEDF compared to healthy controls (*p* = 0.03). There was a statistically significant rise in plasma PEDF for PDR vs. healthy controls (*p* = 0.005) but there was no significant difference between the other diabetic retinopathy groups. While it is premature to form any conclusions, PEDF may not be one of the more promising blood markers of DR; demonstrating that markers in the vitreous may not necessarily translate to reliable markers in the serum or plasma.

There has also been interest in measuring serum levels of neuroprotective factors which have been demonstrated to be reduced in the vitreous in diabetics. Serum levels of brain derived neurotrophic factor (BDNF) were shown to be significantly lower in NPDR and PDR patients compared to healthy controls in a trial analyzing a total of 114 patients (27 NDR, 30 NPDR, 30 PDR, and 27 healthy controls; Kaviarasan et al., 2015). However, the lack of significance in comparison to diabetics with no eye disease again argues against its viability as a screening marker.

Decreased plasma levels of BDNF were also demonstrated to be an independent risk factor in DR in the Chinese population (Li et al., 2002). This study enrolled 344 patients with diabetes, out of whom 93 were found to have DR—46 with non-vision threatening DR, and 47 with vision threatening DR (VTDR) which was defined as PDR and/or diabetic macular edema. This study found that plasma BDNF levels were significantly lower in all DR compared to diabetics with no eye disease (*p* < 0.001) and also among those with VTDR compared to those with no eye disease and non-VTDR (*p* < 0.001). Based on ROC curves, it was suggested that a plasma BDNF level of less than 13.6 ng/mL was suggestive of DR in general (non-VTDR and VTDR) while a level of < 12.4 mg/mL was suggestive of VTDR. As this study did not analyse the non-VTDR as a separate group, it is difficult to make any conclusions on the potential of BDNF as a screening marker for early disease based on the available data.

Recently, there has also been interest in measuring substances whose blood levels are known to be altered as a result of the metabolic derangements that are characteristic of DM in relation to their association with DR to assess their potential as biomarkers. These include folate, homocysteine, and lipoprotein (a).

Serum homocysteine levels were measured in 330 subjects (50 NDR, 63 NPDR, 62 PDR, 80 healthy controls, and 75 randomly selected patients). The homocysteine levels showed a significant elevation between the groups with progressive severity of DR—comparison between controls and NDR, NDR vs. NPDR and NPDR vs. PDR showed significance levels of *p* < 0.001, *p* < 0.001, and *p* < 0.0001, respectively. This seems to indicate that homocysteine may be a promising screening marker of DR (Malaguarnera et al., 2014).

Folate status is typically reduced in diabetic patients, and as folate may influence the DNA stability and integrity as well as affect the methylation patterns in neural tube tissue this may predispose patients to the development of DR. Malaguarnera et al. (2015) analyzed the plasma levels of folate in 231 subjects (96 NDR, 70 NPDR, and 65 PDR). Severity of diabetic retinopathy was associated with lower folic acid and red cell folate levels and a significant difference was observed between the PDR and NPDR groups (*p* < 0.05). However, the lack of a significant difference between NDR and NPDR makes it less likely to be able to be a stand-alone marker of DR.

Serum lipoprotein (a) levels have also been demonstrated to be elevated in patients with DR compared to patients with no DR. In a comparison of 78 diabetics with no eye disease against 67 with DR, elevated levels of Lp(a) were found in 78.3% of those with DR but in only 21.75% of those with no DR (Malaguarnera et al., 2013). This seems unlikely to be a worthwhile marker for further study.

## Novel proteins (Table 5)

A new era in proteomics technologies has opened new vistas in the search for novel biomarkers of DR. The previous sections review studies utilizing known molecules which were postulated as likely to have a role in DR and their levels were then measured by means of quantitative assays (largely ELISA), however proteomics has opened up the possibility of searching for molecules which had not yet been associated with DR but which show altered levels on analysis of biological samples from patients with DR.

**Table 5:** **Summary of selected studies utilizing proteomics technology on various samples to determine novel proteins associated with presence of varying stages of diabetic retinopathy**.

**Sample analyzed**	**Reference/study design**	**Sample size**	**Key findings**	**Method of analysis**
Serum	Kim et al., 2013/cross sectional	*N* = 60 (15 NDR, 15 mild NPDR, 15 moderate NPDR, and 15 severe NPDR)	Twenty-eight candidate proteins were identified that underwent changes in expression with progression of retinopathy. Combinations of four of these proteins were able to distinguish between mild, moderate, and severe NPDR generating AUC values (>0.7) (see main text for details)	Multiple reaction monitoring (MRM) using triple quadrupole LC-MS/MS (4000 QTrap coupled with nano Tempo MDLC, Applied Biosystems)
Serum	Liu et al., 2011/cross sectional	*N* = 32 (8 NDR, 8 NPDR, 8 PDR, 8 healthy controls)	Four low abundance proteins identified (β2-GPI, AHSG, α1-AGP, apo A1); β2-GPI expression increased with progressive severity of DR (NDR vs. NPDR ratio 1.58; NDR vs. PDR ration 1.84) PDR vs. NPDR no significant rise (ratio = 1.17)	2D-DIGE
Tear fluid	Csosz et al., 2012/cross sectional	*N* = 145 (119 diabetics with NDR, NPDR and PDR, 26 healthy volunteers	Six proteins were identified as possible markers of DR in tear fluid (lipocalin 1, lactotransferrin, lacritin, lysozyme C, lipophilin A, and immunoglobulin lambda chain)	Nano HPLC couples with ESI-MS/MS mass spectrometry
Tear fluid	Kim et al., 2012/cross sectional	*N* = 41 (NDR 10, NPDR 17, healthy controls 14)	Twenty proteins were differentially expressed between the study groups, three of which were confirmed by Western Blot (LCN-1, HSP 27, B2M)	ESI-Q-TOF MS/MS

*NDR, diabetic with no retinopathy; NPDR, no proliferative diabetic retinopathy; PDR, proliferative diabetic retinopathy; 2D-DIGE, two dimensional fluorescence difference gel electrophoresis; β2-GPI, beta 2- glycoprotein I, AHSG, alpha2-HS-glycoprotein; α1-AGP, alpha1acid glycoprotein, apo A1, apolipoprotein A1; LCN1, lipocalin 1; HSP 27, heat shock protein 27; B2M, beta-2 microglubulin*.

The initial approach involved using this proteomics technology to analyze the vitreous of patients with PDR (Kim et al., 2007; Shitama et al., 2008). Subsequently comparisons were made between the proteomes of vitreous and serum in non-diabetic controls, NPDR and PDR patients which was able to identify several biomarker candidates for NPDR (Kim et al., 2010). The potential candidate proteins identified were then studied further and 28 candidate proteins were found to undergo changes as patients progressed from mild to moderate NPDR (Kim et al., 2013). Combinations of four markers from these 28 candidate proteins were shown to be able to differentiate between NDR, mild NPDR, and moderate NPDR. Specifically, complement factor H, prothrombin, apolipoprotein A-I, and α-macroglobulin were specifically altered (increased or decreased) preferentially in mild NPDR but did not change in patients with no apparent DR or moderate NPDR. Using logistic regression analysis, these four markers were able to correctly classify 30 cases of mild NPDR from those with no apparent disease with 83.3% accuracy. With reference to moderate NPDR, a combination of four markers (afamin, apolipoprotein C-III, complement factor B, and kallistatin) was able to demonstrate 100% accuracy in differentiating patients with moderate NPDR from those without DR. This is a very encouraging finding and suggests that a panel of these biomarkers may increase the likelihood of being able to screen for and make reasonable estimates of the stage of DR in the population.

An additional novel method utilizing proteomic technology has been the analysis of tear fluid as a potential screening agent for DR. It has been noted that studies using serum to evaluate for biomarkers of DR faced difficulties due to the fact that serum from diabetic patients contained several glycosylated proteins. Tear fluid was postulated to be an accurate and efficient way of screening for DR; furthermore tear fluid is easily obtained through non-invasive methods. Evaluation of the proteome of tear fluid has been found to have significant differences between patients with early DR and healthy controls (Csosz et al., 2012; Kim et al., 2012). An attempt to apply machine learning algorithms to the variable protein concentration of six proteins identified by Csosz et al. (2012) as independent biomarkers of DR in tear fluid was able to obtain 74% sensitivity, 48% specificity and 65% accuracy highlighting the potential of tear fluid as a promising biological sample to screen for DR (Torok et al., 2013).

## MicroRNA (miRNA)

miRNAs are a class of highly conserved 19–25 nucleotide noncoding RNAs that regulate gene expression at the posttranscriptional level. By annealing to partially complementary sequences in the target mRNAs, miRNAs mediate translational repression or degradation of mRNAs, resulting in the downregulation of protein expression (Wu et al., 2012). There is evidence that several miRNAs target specific mRNAs for regulating the progression of DR. These include miRNAs-126, -200b, and -31, all of which are involved in vasculature regulation and therefore are crucial for suppressing angiogenesis in DR. miRNAs-146, -155, -132, and -21 have role in the chronic inflammation that is a key factor in the development of DR (Xiong et al., 2014). Other miRNAs whose level has been shown to be altered in patients with DR but whose role remains to be clearly defined include miR-182, -96, -183, -211, -204, and -124 which were significantly increased during progress of DR, and miR-10b, -10a, 219-2-3p, -144, -338, -199a-3p which were significantly decreased (Wu et al., 2012). Others with altered levels include miR-24, -323, -92a, -369, -219, -203a,-34c, -350, -410, -592, -758, -216a, -351, -137, -935 (upregulated), and miR-375 and -212 which are downregulated (Xiong et al., 2014). Recent work based on a nested case-control study design of 300 samples in two prospective cohorts of the Diabetic Retinopathy Candesartan Trial (DIRECT): PROTECT-1 and PREVENT-1 has identified two angiogenic miRNAs miR-320a and -27b as potential biomarkers for diabetic retinopathy (Zampetaki et al., 2016).

miRNAs are deemed to show great promise as biomarkers. Although the main biological activity of miRNA occurs in the intracellular space, these molecules have been found to be stable in many biological fluids, including human serum, plasma, urine, saliva, tears, aqueous humor, and vitreous humor (Mastropasqua et al., 2014) which makes them potentially detectable in wide variety of easily obtainable clinical specimens. Also, they are very stable and long lived molecules. Once released from cells into circulation, they have a long life span (~≥2 weeks) and they are stable in plasma, serum and urine not only under standard conditions but also after undergoing several freeze-thaw cycles, strong variation in pH, and long exposure to room temperature as well as having efficient recovery and the fact that quantitative assays to measure these molecules are currently available (Mastropasqua et al., 2014; Joglekar et al., 2016).

## Conclusion

At present, a definitive marker for early stage DR or one that can be detected well-before the development of any retinopathy remains elusive. However, the findings of the two prospective studies done with the DCCT cohort have demonstrated promising markers of increased risk for development and progression of DR which should be pursued further. While a large number of other potential markers have been suggested by the other studies reviewed, their cross sectional study design makes the conclusions drawn less robust. An additional consideration from the work reviewed is that the multifactorial etiology of DR makes it likely that a successful screening strategy may require a panel of markers as opposed to utilizing a single marker; also serum markers of DR may not necessarily correspond to markers of DR found in the vitreous.

There are multiple potential confounders that need to be addressed in the search for a screening marker, including geographic, ethnic, and genetic variations in the study populations as well as the varying phenotypes of DR. Large scale, multicenter prospective studies need to be done in order to conclusively determine the reliability of the various biomarkers of early stage DR.

## Author contributions

PP conducted the literature review, analysis, and writing of the manuscript. LLH and KAK provided vital input related to the context. PP and KAK founded the research topic.

## Funding

Monash University Malaysia ECR Grant (Project no. 5140077-000-00) awarded to PP, and PVC Award Research Grant (Project no. PVC-ECR-2016) awarded to LLH.

### Conflict of interest statement

The authors declare that the research was conducted in the absence of any commercial or financial relationships that could be construed as a potential conflict of interest.

## References

[B1] Adams A. J. Bearse M. A. Jr. (2012). Retinal neuropathy precedes vasculopathy in diabetes: a function-based opportunity for early treatment intervention? Clin. Exp. Optom. 95, 256–265. 10.1111/j.1444-0938.2012.00733.x 22497728

[B2] Aiello L. P. Cahill M. T. Wong J. S. (2001). Systemic considerations in the management of diabetic retinopathy. Am. J. Ophthalmol. 132, 760–776. 10.1016/S0002-9394(01)01124-2 11704039

[B3] Amadio M. Bucolo C. Leggio G. Drago F. Govoni S. Pascale A. (2010). The PKCbeta/HuR/VEGF pathway in diabetic retinopathy. Biochem. Pharmacol. 80, 1230–1237. 10.1016/j.bcp.2010.06.033 20599775

[B4] Amadio M. Scapagnini G. Lupo G. Drago F. Govoni S. Pascale A. (2008). PKCbeta/HuR/VEGF: A new molecular cascade in retinal pericytes for the regulation of VEGF gene expression. Pharmacol. Res. 57, 60–66. 10.1016/j.phrs.2007.11.006 18206386

[B5] Boehm B. Lang G. Volpert O. Jehle P. M. Kurkhaus A. Rosinger S.. (2003). Low content of the natural ocular anti-angiogenic agent pigment epithelium-derived factor (PEDF) in aqueous humor predicts progression of diabetic retinopathy. Diabetologia 46, 394–400. 10.1007/s00125-003-1040-9 12687338

[B6] Boehm B. O. Schilling S. Rosinger S. Lang G. E. Lang G. K. Kientsch-Engel R. (2004). Elevated serum levels of *N*-carboxymethyl-lysine, an advanced glycation end product, are associated with proliferative diabetic retinopathy and macular oedema. Diabetologia 47, 1376–1379. 10.1007/s00125-004-1455-y 15258735

[B7] Brownlee M. (2005). The pathobiology of diabetic complications. Diabetes 54, 1615–1625. 10.2337/diabetes.54.6.1615 15919781

[B8] Cavusoglu A. Bilgili S. Alaluf A. Dogan A. Yilmaz F. Aslanca D.. (2007). Vascular endothelial growth factor level in the serum of diabetic patients with retinopathy. Ann. Ophthalmol. (Skokie) 39, 205–208. 10.1007/s12009-007-0037-2 18025626

[B9] Choudhuri S. Dutta D. Sen A. Chowdhury I. H. Mitra B. Mondal L. K.. (2013). Role of *N*-ϵ-carboxy methyllysine, advanced glycation end products and reactive oxygen species for the development of nonproliferative and proliferative retinopathy in type 2 diabetes mellitus. Mol. Vis. 19, 100–113. 23378723 PMC3559098

[B10] Cowan C. Muraleedharan C. K. O'Donnell J. J. I. I. I. Singh P. K. Lum H. Kumar A. (2014). MicroRNA-146 inhibits thrombin- induced NF-KB activation and subsequent inflammatory responses in human retinal endothelial cells. Invest. Ophthalmol. Vis. Sci. 55, 4944–4951. 10.1167/iovs.13-13631 24985472

[B11] Csosz E. Boross P. Csutak A. Berta A. Toth F. Poliska S.. (2012). Quantitative analysis of proteins in the tear fluid of patients with diabetic retinopathy. J. Proteomics 75, 2196–2204. 10.1016/j.jprot.2012.01.019 22300579

[B12] Cunha-Vaz J. Bernardes R. Lobo C. (2011). Blood-retinal barrier. Eur. J. Ophthalmol. 21(Suppl. 6), S3–S9. 10.5301/EJO.2010.6049 23264323

[B13] Cunha-Vaz J. Ribeiro L. Lobo C. (2014). Phenotypes and biomarkers of diabetic retinopathy. Prog. Retin. Eye Res. 41, 90–111. 10.1016/j.preteyeres.2014.03.003 24680929

[B14] Dawson D. Volpert O. Gillis P. Crawford S. Xu H. Benedict W.. (1999). Pigment epithelium-derived factor: a potent inhibitor of angiogenesis. Science 285, 245–248. 10.1126/science.285.5425.245 10398599

[B15] Diabetes Control Complications Trial (DCCT) Research Group (1993). The effect of intensive treatment of diabetes on the development and progression of long-term complications in insulin-dependent diabetes mellitus. N. Engl. J. Med. 329, 977–986. 8366922 10.1056/NEJM199309303291401

[B16] Doganay S. Evereklioglu C. Er H. Turkoz Y. Sevinc A. Mehmet N.. (2002). Comparison of serum NO, TNF-alpha, IL-1beta, sIL-2R, IL-6 and IL-8 levels with grades of retinopathy in patients with diabetes mellitus. Eye 16, 163–170. 10.1038/sj/eye/6700095 11988817

[B17] Dong N. Shi H. Xu B. Cai Y. (2015). Increased plasma S100A12 levels are associated with diabetic retinopathy and prognostic biomarkers of macrovascular events in type 2 diabetic patients. Invest. Ophthalmol. Vis. Sci. 56, 4177–4185. 10.1167/iovs.15-16470 26132777

[B18] Du J. Li R. Xu L. Ma R. Liu J. Cheng J.. (2016). Increased serum chemerin levels in diabetic retinopathy of type 2 diabetic patients. Curr. Eye Res. 41, 114–120. 10.3109/02713683.2015.1004718 25848840

[B19] Du J. Li X. Li R. Xu L. Ma R.-R. Liu S.-F.. (2014). Elevation of serum apelin-13 associated with proliferative diabetic retinopathy in type 2 diabetic patients. Int. J. Ophthalmol. 7, 968–973. 10.3980/j.issn.2222-3959.2014.06.10 25540748 PMC4270990

[B20] Fatima S. Butt Z. Bader N. Pathan A. Hussain S. Iqbal N. (2015). Role of multifunctional chemerin in obesity and preclinical diabetes. Obes. Res. Clin. Prac. 9, 5–8. 10.1016/j.orcp.2015.01.004 25666091

[B21] Ford E. S. Zhao G. Li C. (2010). Pre-diabetes and the risk for cardiovascular disease. J. Am. Coll. Cardiol. 55, 1310–1317. 10.1016/j.jacc.2009.10.060 20338491

[B22] Fosmark D. S. Torjesen P. A. Kilhovd B. K. Berg T. J. Sandvik L. Hanssen K. F.. (2006). Increased serum levels of the specific advanced glycation end product methylgyoxal-derived hydroimidazole are associated with retinopathy in patients with type 2 diabetes mellitus. Metab. Clin. Exp. 55, 232–236. 10.1016/j.metabol.2005.08.017 16423631

[B23] Genuth S. Sun W. Cleary P. Sell D. R. Dahms W. Malone J.. (2005). Glycation and carboxymethyllysine levels in skin collagen predict the risk of future 10-year progression of diabetic retinopathy and nephropathy in the diabetes control and complications trial and epidemiology of diabetes interventions and complications participants with type 1 diabetes. Diabetes 54, 31013–33011. 10.2337/diabetes.54.11.3103 16249432 PMC2622724

[B24] Giet M. V. D. Henkel C. Schuchardt M. Tolle M. (2015). Anti-VEGF drugs in eye diseases: local therapy with potential systemic effects. Curr. Pharm. Des. 21, 1–9. 10.2174/1381612821666150225120314 25714990

[B25] Giurdanella G. Anfuso C. D. Olivieri M. Lupo G. Caporarello N. Eandi C. M.. (2015). Aflibercept, bevacizumab and ranibizumab prevent glucose-induced damage in human retinal pericytes *in vitro* through a PLA2/COX-2/VEGF-A pathway. Biochem. Pharmacol. 96, 278–287. 10.1016/j.bcp.2015.05.017 26056075

[B26] Grossin N. Wautier M. Meas T. Guillausseau P. J. Massin P. Wautier J. (2008). Severity of diabetic microvascular complications is associated with a low soluble RAGE level. Diabetes Metab. 34, 392–395. 10.1016/j.diabet.2008.04.003 18701333

[B27] Hamano K. Nakadaira I. Suzuki J. Gonai M. (2014). *N*-terminal fragment of probrain natriuretic peptide is associated with diabetes microvascular complications in type 2 diabetes. Vasc. Health Risk Manag. 10, 585–595. 10.2147/VHRM.S67753 25328404 PMC4199566

[B28] Hammes H.-P. Lin J. Renner O. Shani M. Lundqvist A. Betsholtz C.. (2002). Pericytes and the pathogenesis of diabetic retinopathy. Diabetes 51, 3107–3112. 10.2337/diabetes.51.10.3107 12351455

[B29] Hiller R. Sperduto R. D. Podgor M. J. Ferris F. L. III. Wilson P. W. (1988). Diabetic retinopathy and cardiovascular disease in type II diabetics. The framingham heart study and the framingham eye study. Am. J. Epidemiol. 128, 402–409. 3293436 10.1093/oxfordjournals.aje.a114980

[B30] Hirata K. Kubo K. (2004). Relationship between blood levels of *N*-Carboxymethyl-lysine and pentosidine and the severity of microangiopathy in Type 2 diabetes. Endocr. J. 51, 537–544. 10.1507/endocrj.51.537 15644571

[B31] Holopigian K. Seiple W. Lorenzo M. Carr R. (1992). A comparison of photopic and scotopic electroretinographic changes in early diabetic retinopathy. Invest. Ophthalmol. Vis. Sci. 33, 2773–2780. 1526726

[B32] Ibrahim A. Tawfik A. Hussein K. Elshafey S. Markand S. Rizk N.. (2015). Pigment epithelium-derived factor inhibits retinal microvascular dysfunction induced by 12/15-lipoxygenase-derived eicosanoids. Biochem. Biophys. Acta 1851, 290–298. 10.1016/j.bbalip.2014.12.017 25562624 PMC4312733

[B33] Jain A. Saxena S. Khanna V. K. Shukla R. K. Meyer C. H. (2013). Status of serum VEGF and ICAM-1 and its association with external limiting membrane and inner segment-outer segment junction disruption in type 2 diabetes mellitus. Mol. Vis. 19, 1760–1768. 23922493 PMC3733909

[B34] Jenkins A. Shang S. X. Gosmanova A. Aston C. Dashti A. Baker M. Z.. (2008). Increased serum pigment epithelium derived factor levels in type 2 diabetes patients. Diabetes Res. Clin. Prac. 82, e5–e7. 10.1016/j.diabres.2008.06.019 18715664 PMC2597065

[B35] Jenkins A. Zhang S. Rowley K. Karschimkus C. Nelson C. Chung J. (2007). Increased serum pigment epithelium derived factor (PEDF) is associated with microvascular complications, vascular stiffness and inflammation in Type 1 Diabetes. Diabet. Med. 24, 1345–1351. 10.1111/j.1464-5491.2007.02281.x 17971181

[B36] Joglekar M. V. Januszewski A. S. Jenkins A. J. Hardikar A. A. (2016). Circulating microRNA biomarkers of diabetic retinopathy. Diabetes 65, 22–24. 10.2337/dbi15-0028 26696637

[B37] Joussen A. M. Poulaki V. Le M. L. Koizumi K. Esser C. Janicki H.. (2004). A central role for inflammation in the pathogenesis of diabetic retinopathy. FASEB J. 18, 1450–1452. 10.1096/fj.03-1476fje 15231732

[B38] Kakehashi A. Inoda S. Mameuda C. Kuroki M. Jono T. Nagai R.. (2008). Relationship among VEGF, VEGF receptor, AGEs, and macrophages in proliferative diabetic retinopathy. Diabetes Res. Clin. Prac. 79, 438–445. 10.1016/j.diabres.2007.10.018 18053608

[B39] Kandarakis S. A. Piperi C. Topouzis F. Papavassiliou A. G. (2014). Emerging role of advanced glycation-end products (AGEs) in the pathobiology of eye diseases. Prog. Retin. Eye Res. 42, 85–102. 10.1016/j.preteyeres.2014.05.002 24905859

[B40] Kaul K. Hodgkinson A. Tarr J. M. Kohner E. M. Chibber R. (2010). Is inflammation a common retinal-renal -nerve pathogenic link in diabetes? Curr. Diabetic. Rev. 6, 294–303. 10.2174/157339910793360851 20594163

[B41] Kaviarasan K. Jithu M. Mulla M. A. Sharma T. Sivasankar S. Das U. N.. (2015). Low blood and vitreal BDNF, LXA4 and altered Th1/Th2 cytokine balance are potential reisk factors for diabetic retinopathy. Metab. Clin. Exp. 64, 958–966. 10.1016/j.metabol.2015.04.005 26004392

[B42] Keech A. Mitchell P. Summanen P. A. O'Day J. Davis T. M. E. Moffitt M. S.. (2007). Effect of fenofibrate on the need for laser treatment for diabetic retinoapathy (FIELD study): a randomised controlled trial. Lancet 370, 1687–1697. 10.1016/S0140-6736(07)61607-9 17988728

[B43] Kerkeni M. Saidi A. Bouzidi H. Yahya S. B. Hammami M. (2012). Elevated serum levels of AGEs, sRAGE, and pentosidine in Tunisian patients with severity of diabetic retinopathy. Microvasc. Res. 84, 378–383. 10.1016/j.mvr.2012.07.006 22835520

[B44] Kim H.-J. Kim P.-K. Yoo H.-S. Kim C.-W. (2012). Comparison of tear proteins between healthy and early diabetic retinopathy patients. Clin. Biochem. 45, 60–67. 10.1016/j.clinbiochem.2011.10.006 22040812

[B45] Kim K. Kim S. J. Han D. Jin J. Yu J. Park K. S.. (2013). Verification of multimarkers for detection of early stage diabetic retinopathy using multiple reaction monitoring. J. Proteome Res. 12, 1078–1089. 10.1021/pr3012073 23368427

[B46] Kim K. Kim S. J. Yu H. G. Yu J. Park K. S. Jang I.-J.. (2010). Verification of biomarkers for diabetic retinopathy by multiple reaction monitoring. J. Proteome Res. 9, 689–699. 10.1021/pr901013d 20020744

[B47] Kim T. Kim S. J. Kim K. Kang U.-B. Lee C. Park K. S.. (2007). Profiling of vitreous proteomes from proliferative diabetic retinopathy and nondiabetic patients. Proteomics 7, 4203–4215. 10.1002/pmic.200700745 17955474

[B48] Kiritoshi S. Nishikawa T. Sonoda K. Kukidome D. Senokuchi T. Matsuo T. (2003). Reactive oxygen species from mitochondria induce cyclooygenase-2 gene expression in human mesangial cells- potential role in diabetic nephropathy. Hypertension 52, 2570–2577. 10.2337/diabetes.52.10.2570 14514642

[B49] Klaassen I. Noorden C. J. F. V. Schlingemann R. O. (2013). Molecular basis of the inner blood-retinal barrier and its breakdown in diabetic macular edema and other pathological conditions. Prog. Retin. Eye Res. 34, 19–48. 10.1016/j.preteyeres.2013.02.001 23416119

[B50] Kovacs B. Lumayag S. Cowan C. Xu S. (2011). microRNAs in early Diabetic retinopathy in streptozocin-induced diabetic rats. Invest Ophthamol. Vis. Sci. 52, 4402–4409. 10.1167/iovs.10-6879 21498619

[B51] Lamparter J. Raum P. Pfeiffer N. Peto T. Hohn R. Elflein H. (2014). Prevalence and associations of diabetic retinopathy in a large cohort of prediabetic subjects: the Gutenberg health study. J. Diabetes Complications 28, 482–487. 10.1016/j.jdiacomp.2014.02.008 24630763

[B52] Li L. Han L. Fu Q. Li Y. Liang Z. Su J. (2012). Formation and inhibition of *N*-ϵ-(Carboxymethyl)lysine in saccharide-lysine model systems during microwave heating. Molecules 17, 12758–12770. 10.3390/molecules171112758 23114613 PMC6268514

[B53] Li Q. Zemel E. Miller B. Perlman I. (2002). Early retinal damage in experimental diabetes: electroretinographical and morphological observations. Exp. Eye Res. 74, 615–625. 10.1006/exer.2002.1170 12076083

[B54] Li T. Hu J. Du S. Chen Y. Wang S. Wu Q. (2014). ERK1/2/COX-2/PGE2 signaling pathway mediates GPR-91 dependent VEGF release in streptozocin-induced diabetes. Mol. Vis. 20, 1109–1121. 25324681 PMC4119234

[B55] Liu S. Y. Du X. F. Ma X. Guo J. L. Lu J. M. Ma L. S. (2016). Low plasma levels of brain derived neurotrophic factor are potential risk factors for diabetic retinopathy in Chinese type 2 diabetic patients. Mol. Cell. Endocrinol. 420, 152–158. 10.1016/j.mce.2015.10.010 26493466

[B56] Liu Y. P. Hu S. W. Wu Z. F. Mei L. X. Lang P. Lu X. H.. (2011). Proteomic analysis of human serum from diabetic retinopathy. Int. J. Ophthalmol. 4, 616–622. 10.3980/j.issn.2222-3959.2011.06.08 22553731 PMC3340797

[B57] Liu Y. Tao L. Fu X. Zhao Y. Xu X. (2013). BDNF protects retinal neurons from hyperglycemia through the TrkB/ERK/MAPK pathway. Mol. Med. Rep. 2013, 1773–1778. 10.3892/mmr.2013.1433 23595279

[B58] Longeras R. Farjo K. Ihnat M. Ma J. X. (2012). A PEDF-derived peptide inhibits retinal neovascularization and blocks mobilization of bone marrow-derived endothelial progenitor cells. Exp. Diabetes Res. 2012:518426. 10.1155/2012/518426 21754923 PMC3132462

[B59] Lupo G. Motta C. Giurdanella G. Anfuso C. D. Alberghina M. Drago F.. (2013). Role of phospholipases A2 in diabetic retinopathy: *In vitro* and *in vivo* studies. Biochem. Pharmacol. 86, 1603–1613. 10.1016/j.bcp.2013.09.008 24076420

[B60] Malaguarnera G. Gagliano C. Bucolo C. Vacante M. Salomone S. Malaguarnera M.. (2013). Lipoprotein(a) serum levels in diabetic patients with retinopathy. Biomed Res. Int. 2013:943505. 10.1155/2013/943505 23862162 PMC3687764

[B61] Malaguarnera G. Gagliano C. Giordano M. Salomone S. Vacante M. Bucolo C.. (2014). Homocysteine serum levels in diabetic patients with non proliferative, proliferative and without retinopathy. BioMed. Res. Int. 2014:191497. 10.1155/2014/191497 24877066 PMC4022262

[B62] Malaguarnera G. Gagliano C. Salomone S. Giordano M. Bucolo C. Pappalardo A.. (2015). Folate status in type 2 diabetic patients with and without retinopathy. Clin. Ophthalmol. 9, 1437–1442. 10.2147/opth.s77538 26300625 PMC4536839

[B63] Mastropasqua R. Toto L. Cipollone F. Santovito D. Carpineto P. Mastropasqua L. (2014). Role of micro RNAs in the modulation of diabetic retinopathy. Prog. Ret. Eye Res. 43, 92–107. 10.1016/j.preteyeres.2014.07.003 25128741

[B64] Meleth A. D. Agron E. Chan C.-C. Reed G. F. Arora K. Byrnes G.. (2005). Serum inflammatory markers in diabetic retinopathy. Invest. Ophthalmol. Vis Sci. 46, 4296–4301. 10.1167/iovs.04-1057 16249511

[B65] Mishra N. Saxena S. Shukla R. K. Singh V. Meyer C. H. Kruzliak P. (2015). Association of serum *N*-ϵ-Carboxy methlylysine with severity of diabetic retinopathy. J. Diabet. Complications 30, 511–517. 10.1016/j.jdiacomp.2015.12.009 26782022

[B66] Mohamed Q. Gillies M. C. Wong T. Y. (2007). Management of diabetic retinopathy: a systematic review. JAMA 298, 902–916. 10.1001/jama.298.8.902 17712074

[B67] Moss S. Klein R. Klein B. (1998). The 14-year incidence of visual loss in a diabetic population. Ophthalmology 105, 998–1003. 10.1016/S0161-6420(98)96025-0 9627648

[B68] Nakamura N. Hasegawa G. Obayashi H. Yamakazi M. Ogata M. Nakano N. (2003). Increased concentration of pentosidine, an advanced glycation end produce, and interleukin-6 in the vitreous of patients with proliferative diabetic retinopathy. Diabetes Res. Clin Prac. 62, 93–101. 10.1016/S0168-8227(03)00109-8 12951277

[B69] Nathan D. Chew E. Christophi C. Davis M. Fowler S. Goldstien B.. (2007). The prevalence of retinopathy in impaired glucose tolerance and recent-onset diabetes in the Diabetes Prevention Program. Diabet. Med. 24, 137–144. 10.1111/j.1464-5491.2007.02043.x 17257275 PMC2267935

[B70] Ng Z. X. Chua K. H. Iqbal T. Kuppusamy U. R. (2013). soluble receptor for advanced glycation end-product (sRAGE)/pentosidine ratio: a potential risk factor determinant for type 2 diabetic retinopathy. Int. J. Mol. Sci. 14, 7480–7491. 10.3390/ijms14047480 23552832 PMC3645698

[B71] Ogata N. Matsuoka M. Matsuyama K. Shima C. Tajika A. Nishiyama T.. (2007). Plasma concentration of pigment epithelium derived factor in patients with diabetic retinopathy. J. Clin. Endocrinol. Metab. 92, 1176–1179. 10.1210/jc.2006-2249 17213275

[B72] Ogata N. Nishikawa M. Nishimura T. Mitsuma Y. Matsumura M. (2002). Unbalanced vitreous levels of pigment derived epithelium-derived factor and vascular endothelial growth factor in diabetic retinopathy. Am. J. Ophthalmol. 134, 348–353. 10.1016/S0002-9394(02)01568-4 12208245

[B73] Ozturk B. T. Bozkurt B. Kerimoglu H. Okka M. Kamis U. Gunduz K. (2009). Effect of serum cytokines and VEGF levels on diabetic retinopathy and macular thickness. Mol. Vis. 15, 1906–1914. 19784389 PMC2751798

[B74] Pachydaki S. Tari S. R. Ma W. Tseng J. J. Sosonuv A. A. Lee S. E.. (2006). Upregulation of RAGE and its ligands in proliferative retinal disease. Exp. Eye Res. 82, 807–815. 10.1016/j.exer.2005.09.022 16364297

[B75] Polat S. B. Ugurlu N. Yulek F. Simavli H. Ersoy R. Cakir B.. (2014). Evaluation of serum fibrinogen, plasminogen, α2-anti-plasmin, and plasminogen Activator Inhibitor levels (PAI) and their correlation with Presence of Retinopathy in patients with type 1 DM. J. Diabetes Res. 2014:317292. 10.1155/2014/317292 24818165 PMC4003747

[B76] Rajab H. A. Baker N. L. Hunt K. J. Klein R. Cleary P. A. Lachin J. (2015). The predictive role of markers on inflammation and endothelial dysfunction on the course of diabetic retinopathy in type 1 diabetics. J. Diabetes Complications 29, 108–114. 10.1016/j.jdiacomp.2014.08.004 25441222 PMC4426877

[B77] Rajamani U. Jialal I. (2014). Hyperglycemia induces toll-like receptor-2 and -4 expression and activity in human microvascular retinal endothelial cells: implications for diabetic retinopathy. J. Diabetes Res. 2014:790902. 10.1155/2014/790902 25610879 PMC4293793

[B78] Reis A. Mateus C. Melo P. Figueria J. Cunha-Vaz J. Castelo-Branco M. (2014). Neuroretinal dysfunction with intact blood-retinal barrier and absent vasculopathy in type 1 diabetes. Diabetes 63, 3926–3937. 10.2337/db13-1673 24947354

[B79] Sasongko M. B. Wong T. Y. Jenkins A. J. Nguyen T. T. Shaw J. E. Wang J. J. (2014). Circulating markers of inflammation and endothelial function, and their relationship to diabetic retinopathy. Diabet. Med. 32, 686–691. 10.1111/dme.12640 25407692

[B80] Sato E. Nagaoka T. Yokota H. Takahashi A. Yoshida A. (2012). Correlation between plasma pentosidine concentrations and retinal hemodynamics in patients with type 2 diabetes. Am. J. Ophthalmol. 153, 903–909. 10.1016/j.ajo.2011.10.020 22265156

[B81] Scuderi S. D'amico A. G. Federico C. Saccone S. Magro G. Bucolo C.. (2015). Different retinal expression patterns of IL-1α, IL-1β, and their receptors in a rat model of type 1 STZ-induced diabetes. J. Mol. Neurosci. 56, 431–439. 10.1007/s12031-015-0505-x 25676032

[B82] Shitama T. Hayashi H. Noge S. Uchio E. Oshima K. Haniu H.. (2008). Proteome profiling of vitreoretinal diseases by cluster analysis. Proteomics Clin. Appl. 2, 1265–1280. 10.1002/prca.200800017 19081814 PMC2600457

[B83] Simo R. Hernandez C. (2014). Neurodegeneration in the diabetic eye: new insights and therapeutic perspectives. Trends Endocrinol. Metab. 25, 23–33. 10.1016/j.tem.2013.09.005 24183659

[B84] Spijkerman A. Dekker J. Nijpels G. Adriaanse M. Kostense P. Ruwaard D.. (2003). Microvascular complications at time of diagnosis of type 2 diabetes are similar among diabetic patients detected by targeted screening and patients newly diagnosed in general practice. Diab. Care. 26, 2604–2608. 10.2337/diacare.26.9.2604 12941726

[B85] Stem M. S. Gardner T. W. (2013). Neurodegeneration in the pathogenesis of diabetic retinopathy: molecular mechanisms and therapeutic implications. Curr. Med. Chem. 20, 3241–3250. 10.2174/09298673113209990027 23745549 PMC4071765

[B86] Tang J. Kern T. S. (2011). Inflammation in diabetic retinopathy. Prog. Ret. Eye Res. 30, 343–358. 10.1016/j.preteyeres.2011.05.002 21635964 PMC3433044

[B87] Tarr J. M. Kaul K. Chopra M. Kohner E. M. Chibber R. (2013). Pathophysiology of diabetic retinopathy. ISRN Ophthalmol. 2013:343560. 10.1155/2013/343560 24563789 PMC3914226

[B88] Torok Z. Peto T. Csosz E. Molnar A. MAroz-Szabo Z. Berat A.. (2013). Tear fluid proteomics multimarkers for diabetic retinopathy screening. BMC Ophthalmol. 13:40. 10.1186/1471-2415-13-40 23919537 PMC3770351

[B89] UK Prospective Diabetes Study Group (1998). Intensive blood-glucose control with sulphonylureas or insulin compared with conventional treatment and risk of complications in patients with type 2 diabetes (UKPDS 33). Lancet 352, 837–853. 10.1016/S0140-6736(98)07019-6 9742976

[B90] Unterlauft J. Claudepierre T. Schmidt M. Muller K. Yafai Y. Wiedemann P.. (2014). Enhanced survival of retinal ganglion cells is mediated by Muller glial cell-derived PEDF. Exp. Eye Res. 127, 206–214. 10.1016/j.exer.2014.08.004 25128578

[B91] Van Hecke M. V. Dekker J. M. Nijpels G. (2005). Inflammation and endothelial dysfunction are associated with retinopathy: the Hoorn Study. Diabetologia 48, 1300–1306. 10.1007/s00125-005-1799-y 15918015

[B92] Villarroel M. Ciudin A. Hernandez C. Simo R. (2010). Neurodegeneration: an early event of diabetic retinopathy. World J. Diabetes 1, 57–64. 10.4239/wjd.v1.i2.57 21537428 PMC3083883

[B93] Wilkinson-Berka J. L. (2006). Angiotensin and diabetic retinopathy. Int. J. Biochem. Cell Biol. 38, 752–765. 10.1016/j.biocel.2005.08.002 16165393

[B94] Williams R. Airey M. Baster H. Forrester J. Kennedy-Martin T. Girach A. (2004). Epidemiology of diabetic retinopathy and macular oedema: a systematic review. Eye 18, 963–983. 10.1038/sj.eye.6701476 15232600

[B95] Wu J.-H. Gao Y. Ren A.-J. Zhao A.-H. Zhong M. Peng Y.-J.. (2012). Altered MicroRNA expression profiles in retinas with diabetic retinopathy. Ophthalmic Res. 47, 195–201. 10.1159/000331992 22156553

[B96] Wu L. Fernandez-Loaiza P. Sauma J. Hernandez-Bogantes E. Masis M. (2013). Classification of diabetic retinopathy and diabetic macular edema. World J. Diabetes 4, 290–294. 10.4239/wjd.v4.i6.290 24379919 PMC3874488

[B97] Xiong F. Du X. Hu J. Li T. Du S. Wu Q. (2014). Altered retinal microRNA expression profiles in early diabetic retinopathy: an *in silico* analysis. Curr. Eye Res. 39, 720–729. 10.3109/02713683.2013.872280 24502381

[B98] Yang H. S. Woo J. E. Lee S. J. Park S. H. Woo J. M. (2014). Elevated plasma pentraxin 3 levels are associated with development and progression of diabetic retinopathy in Korean patients with type 2 diabetes mellitus. Invest. Ophthalmol. Vis. Sci. 55, 5989–5997. 10.1167/iovs.14-14864 25159210

[B99] Yanni S. E. McCollum G. W. Penn J. S. (2010). Genetic deletion of COX-2 diminishes VEGF production in mouse retinal muller cells. Exp. Eye Res. 91, 34–41. 10.1016/j.exer.2010.03.019 20398651 PMC2879458

[B100] Zampetaki A. Willeit P. Burr S. Yin X. Langley S. Kiechl S.. (2016). Angiogenic microRNAs linked to incidence and progression of diabetic retinoapthy in Type 1 diabetes. Diabetes 65, 216–227. 10.2337/db15-0389 26395742

[B101] Zhang H. Jia L. Hou X. Lu J. Lu H. Du J.. (2009). Prevalence of and risk factors associated with diabetic retinopathy in pre-diabetic and diabetic population in Shanghai community. Zhonghua Yi Xue Za Zhi 89, 17949–17952. 19862978

